# Remote Silyl Groups Enhance Hydrolytic Stability and Photocleavage Efficiency in Carbamates for Protein Release

**DOI:** 10.1002/anie.202502376

**Published:** 2025-04-25

**Authors:** Masahiko Yoshimura, Ryuto Sasayama, Takashi Kajiwara, Chihiro Mori, Yusuke Nakasone, Tomoko Inose

**Affiliations:** ^1^ Institute for Integrated Cell‐Material Sciences (WPI‐iCeMS) Institute for Advanced Study Kyoto University Yoshida, Sakyo‐ku Kyoto 606–8501 Japan; ^2^ Department of Chemistry Graduate School of Science Kyoto University Kitashirakawa‐Oiwakecho Sakyo‐ku Kyoto 606–8502 Japan; ^3^ The Hakubi Center for Advanced Research Kyoto University Yoshida, Sakyo‐ku Kyoto 606–8501 Japan; ^4^ JST PRESTO Saitama 332–0012 Japan; ^5^ Department of Synthetic Chemistry and Biological Chemistry Graduate School of Engineering Kyoto University Katsura, Nishikyo‐ku Kyoto 606–8502 Japan

**Keywords:** Photo cleavable molecules, Protein release, Steric protection, β‐silyl effect

## Abstract

Photocleavable molecules are valuable tools for biological studies, enabling spatiotemporal activation of molecular functions within cellular environments. In particular, coumarin‐based photolytic molecules are useful because of their ability to flexibly tune the wavelength of photostimulation through their structural modifications. Ideal photocleavable molecular tools require hydrolytic stability and selective susceptibility to photo stimuli. However, conventional coumarin‐based molecules have not simultaneously achieved both highly efficient photocleavage and hydrolysis resistance. Herein, we proposed a novel molecular design concept that introduces a silyl group into coumarin‐based molecules at a position remote from the photolabile bond, creating an ideal photocleavable molecule for chemical biology tools. The established orbital effect of the remotely introduced silyl group improves the photolysis efficiency of coumarin‐based molecules, while its bulkiness substantially enhances their hydrolytic stability in aqueous environments and under enzymatic conditions. Furthermore, this improvement in molecular functionality contributes to the development of high‐performance protein‐release biomaterials.

## Graphical abstract

Introducing remote silyl groups into coumarin derivatives enhances their hydrolytic stability and photocleavage efficiency. This innovative molecular design enables efficient protein release from solid phase material upon photo irradiation, advancing applications in chemical biology and photomanipulation.

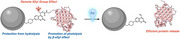

## Introduction

Photoresponsive molecules are widely used in various fields, including materials chemistry and chemical biology.^[^ ^1^, ^2^, ^3^, ^4^, ^5^ ^]^ Such molecules exhibit drastic changes in chemical structure or specific bond cleavages in response to external photostimulation.^[^ ^6^, ^7^ ^]^ For instance, azobenzene, which undergoes *cis–trans* isomerization upon photoirradiation, is a representative photoresponsive chemical structure widely used in photomanipulation techniques.^[^ ^8^ ^]^ Other unique photoresponsive molecules, such as nitrobenzene‐ and coumarin‐linked derivatives, are known as photocleavable molecules, which exhibit specific bond cleavages triggered by photoirradiation.^[^ ^9^, ^10^ ^]^ These photocleavable molecules are valuable tools, particularly in the fields of cell and chemical biology. For instance, caged compounds composed of photocleavable molecules can activate biologically inert signaling molecules via photostimulation. These compounds are useful in light‐stimulated drug delivery systems, enabling highly controlled drug release with precise spatial and temporal resolution.^[^ ^11^, ^12^, ^13^, ^14^, ^15^ ^]^ Furthermore, recent studies have highlighted the usefulness of these photocleavable molecules in applications such as gene editing and optogenetics, enabling precise control of cellular functions.^[^ ^16^, ^17^, ^18^ ^]^ Given this background, there is a strong demand for the development of novel photocleavable molecules that are suitable for biological experiments.

Coumarin‐based photocleavable molecules provide a significant advantage over nitrobenzene‐based derivatives due to their structural flexibility, enabling precise control of absorption wavelength from the ultraviolet (UV) to the near‐infrared (IR) region and broader applicability across experimental setups.^[^ ^19^, ^20^, ^21^, ^22^ ^]^ Typical nitrobenzene‐based photocleavable molecules are difficult to induce bond cleavage at wavelengths longer than 400 nm because of the limited chemical modifications of their benzene ring.^[^ ^23^, ^24^ ^]^ In contrast, coumarin‐based photocleavable molecules, which are relatively easier to modify and have an expandable π structure, allow for the arbitrary selection of wavelengths in the visible light region.^[^ ^10^ ^]^ Despite the advantages of coumarin‐based derivatives, their persistent and widespread use is hindered by lower photocleavage efficiency and potential hydrolytic instability. The photocleavage efficiency of coumarin‐based molecules correlates with the stability of the anionic species generated in the excited state of these molecules under photoirradiation.^[^ ^25^, ^26^ ^]^ Although ester bonds are often incorporated into the structure to stabilize the anionic species,^[^ ^27^, ^28^, ^29^, ^30^ ^]^ they are potentially unstable against hydrolysis and easily degraded in aqueous and cellular environments. On the other hand, Schulte et al. recently reported a novel and effective approach for enhancing the photocleavage efficiency of coumarin‐based derivatives by stabilizing the cation species generated in the excited state.^[^ ^31^ ^]^ They further discussed the escape model of contact ion pairs, which is influenced by environmental conditions and payload size.^[^ ^32^ ^]^ While these studies have provided valuable insights, the structural design of photocleavable chemical tools feasible for cell and chemical biology remains unexplored. New structural guidelines for creating highly functional molecules should considerably elevate the foundation of photomanipulation technologies for flexible applications in cell and chemical biology.

**Figure 1:**
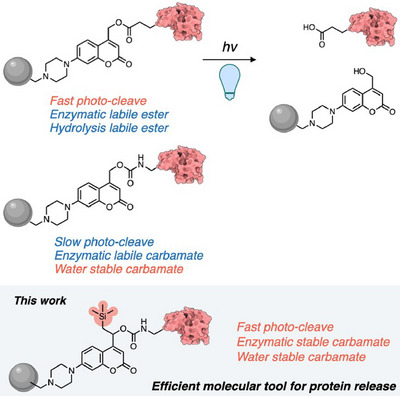
Our research concept: A remotely introduced silyl group sterically protects the hydrolysis‐sensitive carbonyl bond and enhances the photo‐induced bond cleavage reaction.

In this study, we developed a novel coumarin‐based photocleavable molecule that exhibits both effective photocleavage properties and high hydrolysis resistance. Our approach involved introducing silyl groups into coumarin‐based molecules to achieve steric protection of carbonyl bonds and stabilization of cationic species in the excited state (Figure 1). Incorporating reactive functional groups into the molecular structure can impair overall activity. An important molecular design guideline for chemical biology tools is that they should be sufficiently stable in cellular environments and respond selectively and rapidly to desired stimuli.^[^ ^33^ ^]^ Considering that the intracellular environment is aqueous, incorporating chemically unstable structures prone to hydrolysis is impractical.^[^ ^34^, ^35^ ^]^ Additionally, these molecules must exhibit high resistance to metabolic enzymes (both oxidative and hydrolytic),^[^ ^36^ ^]^ reactive chemical species such as glutathione,^[^ ^37^ ^]^ and reactive oxygen species^[^ ^38^ ^]^ present at high concentrations in cells. With these constraints in mind, we designed a coumarin‐based photocleavable molecule with a chemically stable trimethylsilyl (TMS) group. The remotely introduced TMS group sterically protects the hydrolysis‐sensitive carbonyl bond and simultaneously stabilizes the photoexcited cationic species through the β‐silyl effect,^[^ ^39^ ^]^ thereby enhancing photocleavage efficiency. Further, we applied our design to create a biomaterial that enables the photoinduced cleavage of proteins from a solid phase material under low‐intensity light conditions.

## Results and Discussion

### Synthesis and Molecular Properties

**Figure 2:**
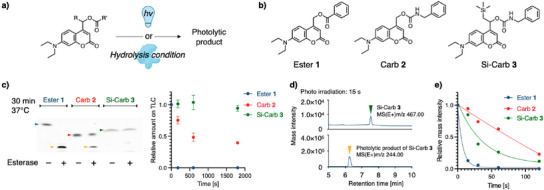
Photo cleavage efficiencies and hydrolytic stabilities of the synthetic coumarin analogues. a) Reaction scheme for the photolytic or hydrolytic conversion of coumarins. The protein illustration was created with BioRender.com. b) Chemical structure of Ester **1**, Carb **2**, Si‐Carb **3**. c) Time‐dependent enzymatic hydrolysis of coumarin analogs. Carboxylesterase‐dependent decrease of coumarin analogs was monitored by TLC and plotted against time (*n* = 3, error bar indicates SD.) Blue, red, green, yellow arrows indicate Ester **1**, Carb **2**, Si‐Carb **3**, photolytic product, respectively. d) HPLC‐MS analysis of Si‐Carb **3** (green arrow) and its photolytic product (yellow arrow). e) Time‐dependent photolysis of coumarin analogs.

To validate our molecular design concept, we synthesized photocleavable coumarin derivatives bearing silyl groups and examined their hydrolytic resistances and photocleavage efficiencies. We synthesized compound **3** (denoted as Si‐Carb **3**) with TMS groups located adjacent to the photolytic cleavage site, as well as the unmodified reference compound **2**, denoted as Carb **2**. In addition to the carbamate version of coumarin derivatives, we synthesized the ester version of coumarins **1** (also denoted as Ester **1**) as the most widely used reference compound (Figure 2a,b).

First, we evaluated the hydrolytic stability of each compound under aqueous conditions and an enzymatic condition. We prepared aqueous solutions of the compounds (10 µM, 0.1% DMSO) at various pH conditions or in the presence of carboxylesterase. Subsequently, we monitored the time‐dependent hydrolysis of the compounds using thin‐layer chromatography (TLC).^[^ ^40^ ^]^ While the ester compound was gradually hydrolyzed at higher pH levels, carbamate compounds were stable under such conditions (Figure S31). The most labile ester, **1**, was fully converted into the corresponding hydrolyzed product after only one min of incubation with carboxylesterase (Figure 2c). Carbamate analog **2** was also hydrolyzed by carboxylesterase, but the hydrolysis reaction was substantially slower than that of Ester **1**. Notably, silyl‐substituted carbamate **3** was far more stable against enzymatic hydrolysis than **1** and **2**. The hydrolytic product of carbamate **3** was not observed until 2.5 h after incubation. Our results are consistent with those of a previous report, suggesting that steric protection around carbonyl bonds can enhance hydrolytic stability because of the introduction of bulky substituents.^[^ ^35^ ^]^

Next, we quantitatively evaluated the time‐dependent photolysis of each compound using high‐performance liquid chromatography‐mass spectrometer (HPLC‐MS) (Figure 2d,e). To assess photolysis of the coumarin derivatives, we irradiated 405 nm light (6.6 mW cm^−2^) to the aqueous solutions of each compound, then monitored the abundance of each compound by detecting the signal intensity at target mass using HPLC‐MS. In all compounds, we observed a time‐dependent decrease in the mass signal intensity of the starting material upon irradiation, accompanied by an increase in the corresponding photolytic products (Figures S33–S35). Ester **1** exhibited faster photolysis than the corresponding carbamate analog **2** because the resulted anionic species from **1** should be further stabilized compared to **2**.^[^ ^25^ ^]^ Notably, we observed a rapid decrease in the mass signal intensity of silyl‐substituted carbamate **3** compared to **2**. This result strongly suggests that the remote silyl group on **3** stabilizes the excited cation species through the β‐silyl effect, which contributes to the acceleration of photolysis.

Thus, we successfully demonstrated that our molecular design featuring a remote silyl group enhanced both hydrolysis resistance and photolytic efficiency through steric protection and established β‐silyl orbital effect on cation stabilization, respectively.

### PhotoLySIS of Coumarins

**Figure 3:**
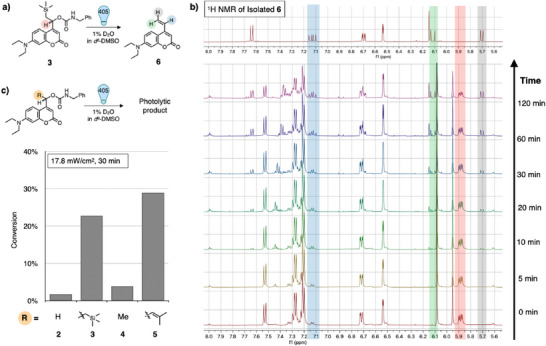
Analysis of the photo‐induced degradation of coumarin analogs using ^1^H NMR spectroscopy. a) Photolytic reaction of silyl substituted coumarin **3**. b) Photo‐induced signal changes of silyl‐substituted coumarin **3** observed using ^1^H NMR spectroscopy. c) Comparison of the photolytic efficiencies of structurally different coumarin analogs.

We further investigated the effects of the substituent on photolysis using ^1^H NMR spectroscopy. We synthesized several derivatives and found that the proton signals changed significantly due to bond cleavage after photo irradiation (Figure 3a,b). To evaluate the impact of the β‐silyl effect on photocleavage efficiency, a methyl‐substituted analog (Me‐Carb **4**) was synthesized and compared with Carb **2** and Si‐Carb **3** (Figure 3c). The photolysis of Me‐Carb **4** was slightly faster than that of unmodified Carb **2**. This reflects the difference in cation stability between the primary and secondary carbocations in the excited state. We observed a significant improvement in photolysis from Me‐Carb **4** to Si‐Carb **3**. This difference between **4** and **3** can be attributed to the β‐cation stabilizing effect of the remote silyl groups. Next, we synthesized vinyl‐derivative **5**, recently reported as a superior photocleavable coumarin designed using an alternative cation‐stabilizing approach, to compare it with our newly developed coumarin. Si‐Carb **3** exhibited photolytic activity comparable to that of the previous reported vinyl‐derivative **5**. In addition, Si‐Carb **3** exhibited substantially greater stability against enzymatic hydrolysis than the vinyl‐derivative **5**, which can be attributed to the three‐dimensional bulkiness of the TMS group that more effectively protects carbonyl bonds against carboxylesterase (Figure S32).

Intriguingly, we discovered a non‐typical photolytic reaction unique to Si‐Carb **3**. Time‐dependent photolytic analysis using HPLC‐MS tracked a decrease in the mass signal of Si‐Carb **3**, accompanied by the emergence of a new mass signal corresponding to photolytic products (Figures 2d,e, S37). The predominant new signal originated from a photolytic product differ from the typical alcohol‐type photolytic product. Structural identification through NMR spectroscopy and mass spectrometry confirmed that this non‐typical photolytic conversion of Si‐Carb **3** resulted in the formation of vinyl coumarin **6** (Figure 3b). We hypothesize that the formation of vinyl coumarin **6** involves desilylation during or after photolysis, potentially, proceeding via hydrolysis, intramolecular silylcarbonylation or Peterson‐type desilylation. Plausible mechanisms for these reactions are depicted in Figure S39, including both stepwise pathways involving intermediate species and concerted processes. Although further mechanistic studies are necessary to distinguish between these possibilities, a combination of time‐resolved analysis of photolytic intermediates and computational studies has the potential to provide deeper insights into the underlying photolysis pathways.

### Photophysical Properties of Coumarins

**Figure 4:**
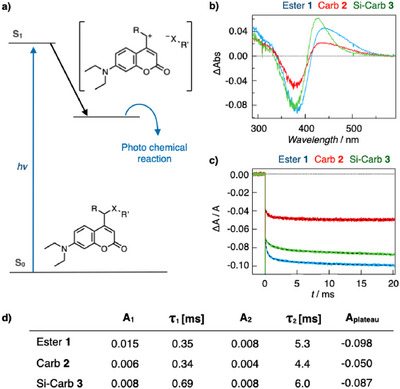
The TA signals of coumarins. a) Schematic representation of possible coumarin photolysis mechanism after excitation. b) TA spectra at 25 ms after excitation. c) TA signals monitored at 385 nm. The signals are analyzed by a bi‐exponential function, with the fitting curves shown as black dashed lines. d) Summary of kinetic parameters derived from TA signal fitting.

The remote effect of the silyl group in stabilizing excited cation species was directly examined using transient absorption (TA) spectroscopy. The photoinduced cleavage mechanism of coumarins has been extensively studied, and the heterolytic dissociation of the C─X bond via a singlet excited state, leading to ion pair formation on an ultrafast timescale, is widely accepted (Figure 4a).^[^ ^25^, ^26^, ^32^, ^41^, ^42^ ^]^ Here, we focused on determining reaction yields and detecting processes following ion pair formation by monitoring delayed TA signals. The TA spectra measured with a 25 ms delay revealed key features: a positive absorption below 330 nm, a negative peak at 385 nm, and a positive peak at 450 nm (Figure 4b). A previous study investigating the photolysis mechanism of coumarin derivatives reported similar TA spectral characteristics,^[^ ^42^ ^]^ indicating that the observed features correspond to the photolytic cleavage process of coumarin‐based compounds. The negative peak at 385 nm represents the ground‐state bleach of the coumarin chromophore, while the positive peaks at 330 and 450 nm are attributed to the formation of the photolytic compounds of coumarins. Despite differences in intensity, the spectra of Ester 1 and Carb 2 showed similar shapes, suggesting the formation of identical photoproducts. In contrast, Si‐Carb **3** exhibited distinct features, including a shifted absorption peak. This observation is potentially associated with a unique photolytic reaction pathway of Si‐Carb **3**, resulting in the production of vinyl coumarin **6**, as supported from the HPLC‐MS and NMR analyses. To analyze reaction kinetics, TA signals were recorded at 385 nm for compounds **1**–**3** (Figure 4c). A fluorescence spike and a rapid absorption decrease were observed within the instrument's response time, with the latter indicating ion pair formation. This was followed by decays on the millisecond timescale, which subsequently plateaued. The TA signals were analyzed by a bi‐exponential function [Eq. (S1) in the Supporting information], with the resulting rate constants and amplitudes summarized in Figure 4d. Notably, the rate constants showed minimal variation among the samples, indicating common underlying mechanisms despite the unique spectral behavior of Si‐Carb **3**. These processes are attributed to structural rearrangements and dissociation reactions. The absorption change at the plateau (A_plateau_) followed the order Ester **1** > Si‐Carb **3 **> Carb **2**, aligning with reaction yields determined by HPLC‐MS. The higher yield of Si‐Carb **3** compared to Carb **2** highlights the stabilizing excited cation species influenced by the β‐silyl effect.

### Synthesis and Application as a Chemical Biology Tool

**Figure 5:**
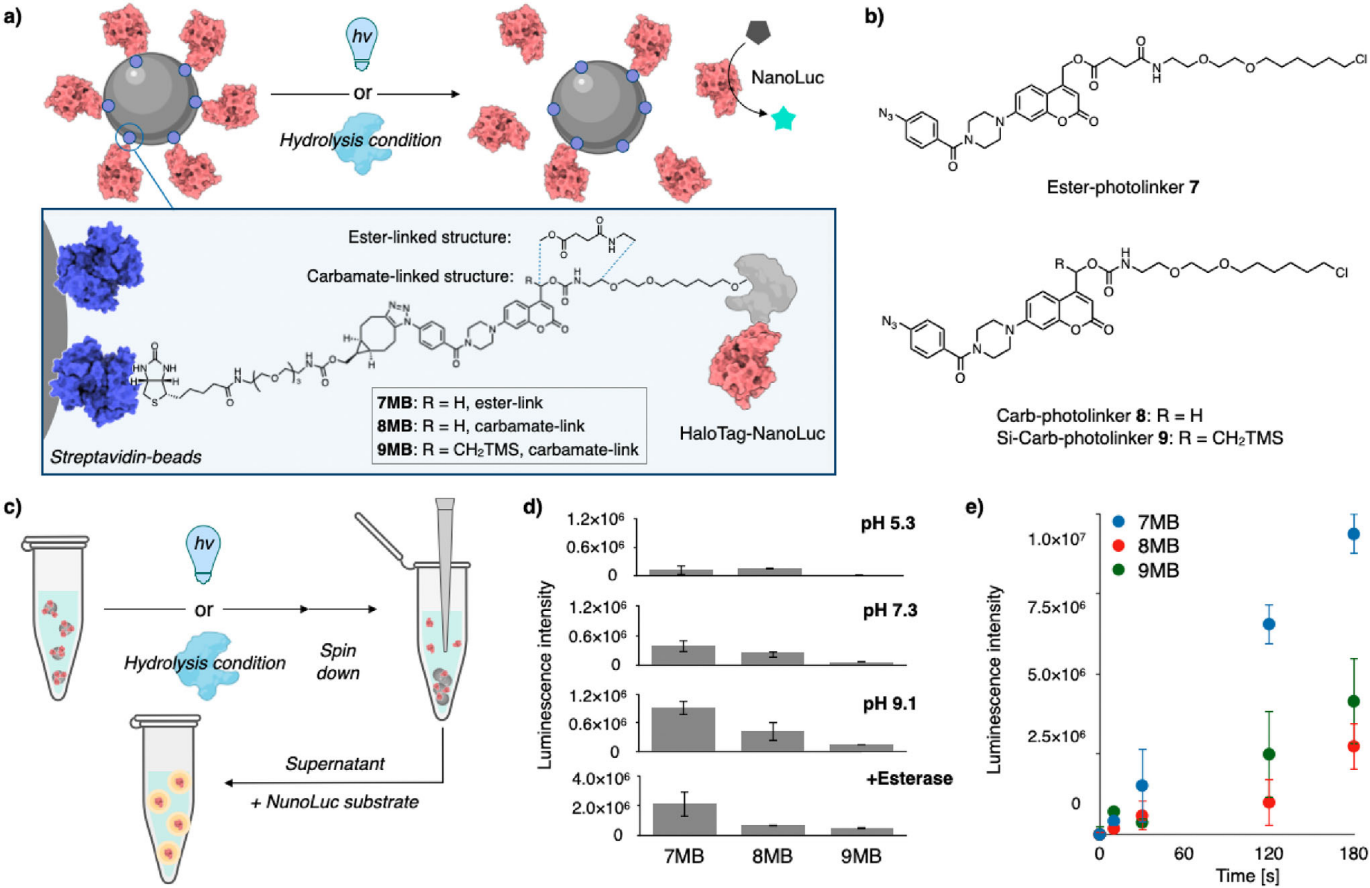
Photo‐induced protein release experiments using photolinkers based on our developed coumarin derivatives. a) Reaction scheme for the photolytic or hydrolytic release of luciferase, NanoLuc, from a solid material. The protein illustration was created using BioRender.com. b) Chemical structures of photolinkers **7**–**9**. c) Schematic representation of the protein releasing experiments. d) Hydrolysis stability under different pH conditions or in the presence of carboxylesterase. The hydrolytic release of NanoLuc was monitored by detecting luminescence intensities at the different pH of the supernatant solution (*n* = 3, error bar indicates SD). e) Photo‐induced protein release. Photo‐induced release of NanoLuc was monitored by detecting luminescence intensities in the supernatant solution (*n* = 3, error bar indicates SD).

The photocleavable molecules using the chemically stable remote silyl group exhibited ideal molecular properties for chemical biology research and cellular experiments. We further utilized our developed photocleavable structure to create biomaterials enabling an efficient protein release. To achieve the desired molecular functionality for protein‐releasing materials, we designed string‐like photocleavable molecules termed photolinkers, with one end designed to bind to the protein of interest and the other end designed to bind to the solid phase material (Figure 5a). To connect proteins with photolinkers, we selected the HaloTag conjugation technology and installed a HaloTag binding ligand, hexyl chloride, on one end of the photolinkers. For binding to the solid phase material, the other end was designed to incorporate an azide group, allowing custom structural modifications suitable for diverse targets using click chemistry (Figure 5b). We prepared three types of photolinkers by stepwise chemical synthesis (Figure 5b, Scheme S2), namely non‐decorated ester‐linked coumarin (Ester‐photolinker **7**), non‐decorated carbamate‐linked coumarin (Carb‐photolinker **8**), and silyl‐substituted carbamate‐linked coumarin (Si‐Carb‐photolinker **9**), to investigate and compare the effects of the remote silyl group. HaloTag‐fused proteins were tethered to magnetic beads using the synthesized photolinkers (Figure 5a, Scheme S3). Initially, we reacted an excess of photolinkers with commercially available biotin‐BCN to quantitatively obtain the click product. Subsequently, excess HaloTag fusion protein was reacted with a biotinylated photolinker solution, successfully creating a molecule that links biotin and the HaloTag fusion protein via a photocleavable coumarin structure. SDS‐PAGE was used to verify the desired conjugation by detecting coumarin‐derived fluorescence in the HaloTag fusion protein band (Figure S40). To facilitate the detection of protein release efficiency, a luciferase called NanoLuc^[^ ^43^ ^]^ was selected as a model protein and used in the HaloTag fusion protein.^[^ ^44^ ^]^ Finally, the resulting conjugates were immobilized on streptavidin‐binding magnetic beads to produce the photoresponsive biomaterial (**7MB**‐**9** **MB**).

We investigated the hydrolytic resistance and photorelease activity of these materials and found that their properties reflected the functionality of the photocleavable coumarin structure. We dispersed the photoresponsive biomaterial in aqueous solutions with various pH values and measured the luminescence of NanoLuc released from the solid phase material via hydrolysis (Figure 5c). Among our biomaterials, **7** **MB** was the most susceptible to the hydrolysis. We observed a significantly slower protein release from **9** **MB** using Si‐Carb‐photolinker compared to **8** **MB** (Figure 5d). Next, we evaluated the hydrolytic release of the proteins in the presence of carboxylesterase. In this study, minimal release of NanoLuc from the solid phase material was observed when Si‐Carb‐photolinker **9** was utilized (Figure 5d). These results, which directly reflect the hydrolytic stability of the photolinkers, are consistent with the molecular functionality of coumarins (Figures 2c, S31). Similar outcomes were obtained for photolysis (Figure 5e). The biomaterial **9** **MB** bearing Si‐Carb‐photolinker **9** efficiently released NanoLuc upon photoirradiation compared with **8** **MB** bearing Carb‐photolinker **8**. Altogether, we demonstrated that the improvement in molecular functionality contributes to the creation of high‐performance protein‐release biomaterials.

## Conclusion

We report the development of a high‐performance photocleavable molecular tool for chemical biology research based on the concept of remote silyl group introduction. This approach allowed the design of a photocleavable coumarin derivative with both high hydrolysis resistance and efficient photolytic performance.

In addition, our molecular design contributed to the successful fabrication of photo‐responsive protein‐releasing materials. Molecular tools incorporating this photocleavable structure have demonstrated enhanced performance in processes involving the immobilization and light‐induced release of target proteins in solid phase materials. We anticipate that our newly developed coumarin will elevate the fundamentals of photo‐manipulation technology, thereby contributing to technological innovation across diverse fields such as biology, materials science, chemical synthesis.

## Supporting Information

Supplementary tables and figures, full experimental procedures and analytical data (^1^H and ^13^C NMR and HR‐MS spectral data) for new compounds, photochemical data, HPLC‐MS data, gel electrophoresis data. (PDF) The authors have cited additional references within the Supporting Information.^[^ ^45^, ^46^ ^]^

## Author Contributions

M.Y. and T.I. directed the project. R.S. and M.Y. synthesized the small molecules. C.M. prepared the recombinant proteins. T.K. and M.Y. conducted HPLC‐MS analysis. Y.N. and T.I. analyzed photo‐chemical properties of synthetic molecules. M.Y. and T.I. conducted biochemical experiments and analysis. The manuscript was written through contributions of Y.N., M.Y., and T.I. All authors have given approval to the final version of the manuscript.

## Conflict of Interests

The authors declare no conflict of interest.

## Supporting information



Supporting Information

## Data Availability

The data that support the findings of this study are available from the corresponding author upon reasonable request.

## References

[1] G. Mayer , A. Heckel , Angew. Chem. Int. Ed. 2006, 45, 4900–4921. 10.1002/anie.200600387 16826610

[2] C. Brieke , F. Rohrbach , A. Gottschalk , G. Mayer , A. Heckel , Angew. Chem. Int. Ed. 2012, 51, 8446–8476. 10.1002/anie.201202134 22829531

[3] E. R. Ruskowitz , C. A. DeForest , Nat. Rev. Mater. 2018, 3, 17087.

[4] I. M. Welleman , M. W. H. Hoorens , B. L. Feringa , H. H. Boersma , W. Szymański , Chem. Sci. 2020, 11, 11672–11691. 34094410 10.1039/d0sc04187d PMC8162950

[5] Y. Deng , G. Long , Y. Zhang , W. Zhao , G. Zhou , B. L. Feringa , J. Chen , Light Sci. Appl. 2024, 13, 63. 38429259 10.1038/s41377-024-01391-8 PMC10907585

[6] H. M. Bandara , S. C. Burdette , Chem. Soc. Rev. 2012, 41, 1809–1825. 22008710 10.1039/c1cs15179g

[7] R. Weinstain , T. Slanina , D. Kand , P. Klán , Chem. Rev. 2020, 120, 13135–13272. 33125209 10.1021/acs.chemrev.0c00663 PMC7833475

[8] I. C. D. Merritt , D. Jacquemin , M. Vacher , Phys. Chem. Chem. Phys. 2021, 23, 19155–19165. 34195720 10.1039/d1cp01873f

[9] P. Klán , T. Šolomek , C. G. Bochet , A. Blanc , R. Givens , M. Rubina , V. Popik , A. Kostikov , J. Wirz , Chem. Rev. 2013, 113, 119–191. 23256727 10.1021/cr300177k PMC3557858

[10] M. J. Hansen , W. A. Velema , M. M. Lerch , W. Szymanski , B. L. Feringa , Chem. Soc. Rev. 2015, 44, 3358–3377. 25917924 10.1039/c5cs00118h

[11] G. C. R. Ellis‐Davies , Nat. Methods 2007, 4, 619–628. 17664946 10.1038/nmeth1072 PMC4207253

[12] W. Zhao , Y. Zhao , Q. Wang , T. Liu , J. Sun , R. Zhang , Small 2019, 15, e1903060. 31599125 10.1002/smll.201903060

[13] I. Elamri , C. Abdellaoui , J. K. Bains , K. F. Hohmann , S. L. Gande , E. Stirnal , J. Wachtveitl , H. Schwalbe , J. Am. Chem. Soc. 2021, 143, 10596–10603. 34236854 10.1021/jacs.1c02817

[14] L. Tapia , I. Alfonso , J. Solà , Org. Biomol. Chem. 2021, 19, 9527–9540. 34668919 10.1039/d1ob01737c

[15] B. M. Vickerman , E. M. Zywot , T. K. Tarrant , D. S. Lawrence , Nat. Rev. Chem. 2021, 5, 816–834. 37117665 10.1038/s41570-021-00326-w PMC8493544

[16] M. Honda , R. Kimura , A. Harada , K. Maehara , K. Tanaka , Y. Ohkawa , S. Oki , STAR Protoc 2022, 3, 101346. 35496796 10.1016/j.xpro.2022.101346 PMC9046621

[17] C. Rebelo , T. Reis , J. Guedes , C. Saraiva , A. F. Rodrigues , S. Simões , L. Bernardino , J. Peça , S. L. C. Pinho , L. Ferreira , Nat. Commun. 2022, 13, 4135. 35840564 10.1038/s41467-022-31791-6 PMC9287341

[18] P. Kashyap , S. Bertelli , F. Cao , Y. Kostritskaia , F. Blank , N. A. Srikanth , C. Schlack‐Leigers , R. Saleppico , D. Bierhuizen , X. Lu , W. Nickel , R. E. Campbell , A. J. R. Plested , T. Stauber , M. J. Taylor , H. Ewers , Nat. Methods 2024, 21, 666–672. 38459384 10.1038/s41592-024-02204-x PMC11009104

[19] Q. Lin , C. Bao , S. Cheng , Y. Yang , W. Ji , L. Zhu , J. Am. Chem. Soc. 2012, 134, 5052–5055. 22394079 10.1021/ja300475k

[20] Q. Lin , L. Yang , Z. Wang , Y. Hua , D. Zhang , B. Bao , C. Bao , X. Gong , L. Zhu , Angew. Chem. Int. Ed. 2018, 57, 3722–3726. 10.1002/anie.201800713 29446517

[21] W. Qiu , M. Li , Y. Yang , Z. Li , K. Dietliker , Polym. Chem. 2020, 11, 1356–1363.

[22] W. Qiu , C. Gehre , J. P. Nepomuceno , Y. Bao , Z. Li , R. Müller , X.‐H. Qin , Angew. Chem. Int. Ed. 2024, 63, e202404599. 10.1002/anie.202404599 39023389

[23] S. V. Wegner , O. I. Sentürk , J. P. Spatz , Sci. Rep. 2015, 5. 10.1038/srep18309 PMC4680943 26670693

[24] M. P. O'hagan , Z. Duan , F. Huang , S. Laps , J. Dong , F. Xia , I. Willner , Chem. Rev. 2023, 123, 6839–6887. 37078690 10.1021/acs.chemrev.3c00016 PMC10214457

[25] R. Schmidt , D. Geissler , V. Hagen , J. Bendig , J. Phys. Chem. A 2005, 109, 5000–5004. 16833851 10.1021/jp050581k

[26] R. Schmidt , D. Geissler , V. Hagen , J. Bendig , J. Phys. Chem. A 2007, 111, 5768–5774. 17564421 10.1021/jp071521c

[27] A. Gandioso , M. Palau , A. Nin‐Hill , I. Melnyk , C. Rovira , S. Nonell , D. Velasco , J. García‐Amorós , V. Marchán , ChemistryOpen 2017, 6, 375–384. 28638770 10.1002/open.201700067 PMC5474652

[28] G. Bassolino , C. Nançoz , Z. Thiel , E. Bois , E. Vauthey , P. Rivera‐Fuentes , Chem. Sci. 2018, 9, 387–391. 29629108 10.1039/c7sc03627b PMC5868312

[29] L. Josa‐Culleré , A. Llebaria , ChemPhotoChem 2021, 5, 296–314.

[30] J. R. Muralidhar , K. Kodama , T. Hirose , Y. Ito , M. Kawamoto , Polym. J. 2022, 54, 191–198.

[31] A. M. Schulte , G. Alachouzos , W. Szymański , B. L. Feringa , J. Am. Chem. Soc. 2022, 144, 12421–12430. 35775744 10.1021/jacs.2c04262 PMC9284546

[32] A. M. Schulte , G. Alachouzos , W. Szymanski , B. L. Feringa , Chem. Sci. 2024, 15, 2062–2073. 38332822 10.1039/d3sc05725a PMC10848663

[33] P. J. LeValley , R. Neelarapu , B. P. Sutherland , S. Dasgupta , C. J. Kloxin , A. M. Kloxin , J. Am. Chem. Soc. 2020, 142, 4671–4679. 32037819 10.1021/jacs.9b11564 PMC7267699

[34] H. J. Braddick , W. J. Tipping , L. T. Wilson , H. S. Jaconelli , E. K. Grant , K. Faulds , D. Graham , N. C. O. Tomkinson , Anal. Chem. 2023, 95, 5369–5376. 36926851 10.1021/acs.analchem.2c05708 PMC10061367

[35] A. K. Yadav , Z. Zhao , Y. Weng , S. H. Gardner , C. J. Brady , O. D. Pichardo Peguero , J. Chan , J. Am. Chem. Soc. 2023, 145, 1460–1469. 36603103 10.1021/jacs.2c12984 PMC10120059

[36] J. Dong , E. Fernández‐Fueyo , F. Hollmann , C. E. Paul , M. Pesic , S. Schmidt , Y. Wang , S. Younes , W. Zhang , Angew. Chem. Int. Ed. 2018, 57, 9238–9261. 10.1002/anie.201800343 PMC6099261 29573076

[37] H. J. Forman , H. Zhang , A. Rinna , Mol. Asp. Med. 2009, 30, 1–12. 10.1016/j.mam.2008.08.006 PMC2696075 18796312

[38] H. Sies , V. V. Belousov , N. S. Chandel , M. J. Davies , D. P. Jones , G. E. Mann , M. P. Murphy , M. Yamamoto , C. Winterbourn , Nat. Rev. Mol. Cell Biol. 2022, 23, 499–515. 35190722 10.1038/s41580-022-00456-z

[39] E. Block , A. J. Yencha , M. Aslam , V. Eswarakrishnan , J. Z. Luo , A. Sano , J. Am. Chem. Soc. 1988, 110, 4748–4753.

[40] N. Mac Fhionnlaoich , S. Ibsen , L. A. Serrano , A. Taylor , R. Qi , S. Guldin , J. Chem. Educ. 2018, 95, 2191–2196.

[41] C. Hamerla , C. Neumann , K. Falahati , J. von Cosel , L. van Wilderen , M. S. Niraghatam , D. Kern‐Michler , N. Mielke , M. Reinfelds , A. Rodrigues‐Correia , A. Heckel , J. Bredenbeck , I. Burghardt , Phys. Chem. Chem. Phys. 2020, 22, 13418–13430. 32515438 10.1039/c9cp07032j

[42] H. D. Nguyen , M. Abe , J. Am. Chem. Soc. 2024, 146, 10993–11001. 38579283 10.1021/jacs.4c02880

[43] M. P. Hall , J. Unch , B. F. Binkowski , M. P. Valley , B. L. Butler , M. G. Wood , P. Otto , K. Zimmerman , G. Vidugiris , T. Machleidt , M. B. Robers , H. A. Benink , C. T. Eggers , M. R. Slater , P. L. Meisenheimer , D. H. Klaubert , F. Fan , L. P. Encell , K. V. Wood , ACS Chem. Biol. 2012, 7, 1848–1857. 22894855 10.1021/cb3002478 PMC3501149

[44] G. V. Los , L. P. Encell , M. G. McDougall , D. D. Hartzell , N. Karassina , C. Zimprich , M. G. Wood , R. Learish , R. F. Ohana , M. Urh , D. Simpson , J. Mendez , K. Zimmerman , P. Otto , G. Vidugiris , J. Zhu , A. Darzins , D. H. Klaubert , R. F. Bulleit , K. V. Wood , ACS Chem. Biol. 2008, 3, 373–382. 18533659 10.1021/cb800025k

[45] A. Hofer , G. S. Cremosnik , A. C. Müller , R. Giambruno , C. Trefzer , G. Superti‐Furga , K. L. Bennett , H. J. Jessen , Chem. ‐ Eur. J. 2015, 21, 10116–10122. 26033174 10.1002/chem.201500838

[46] J. I. Müller , K. Kusserow , G. Hertrampf , A. Pavic , J. Nikodinovic‐Runic , T. A. M. Gulder , Org. Biomol. Chem. 2019, 17, 1966–1969. 30357251 10.1039/c8ob02273a

