# Supplementary material for: Remote Silyl Groups Enhance Hydrolytic Stability and Photocleavage Efficiency in Carbamates for Protein Release
Source: Angew Chem Int Ed Engl. 2025 Apr 25;64(25):e202502376. doi: 10.1002/anie.202502376 (PMC12171346; doi:10.1002/anie.202502376)
Supplement: Supplementary file 1 — Supporting Information [file ANIE-64-e202502376-s001.docx]

Supporting Information

**Remote Silyl Groups Enhance Hydrolytic Stability and Photocleavage Efficiency in Carbamates for Protein Release**

Masahiko Yoshimura,*^a^ Ryuto Sasayama,^a^ Takashi Kajiwara,^a^ Chihiro Mori,^a^ Yusuke Nakasone,^b^ Tomoko Inose*^acde^

^a^ Institute for Integrated Cell-Material Sciences (WPI-iCeMS),

Kyoto University, iCeMS Research Bldg, Yoshida, Sakyo-ku, Kyoto 606-8501, Japan.

^b^ Department of Chemistry, Graduate School of Science Kyoto University,

Kitashirakawa-Oiwakecho, Sakyo-ku Kyoto 606-8502, Japan

^c^ The Hakubi Center for Advanced Research,

Kyoto University, Kitashirakawa-Oiwakecho, Sakyo-ku Kyoto 606-8502, Japan

^d^ JST PRESTO, Saitama 332-0012, Japan

^e^ Department of Synthetic Chemistry and Biological Chemistry,

Graduate School of Engineering, Kyoto University, Katsura, Nishikyo-ku, Kyoto 615-8501, Japan

* Corresponding author
E-mail: [yoshimura.masahiko.8m@kyoto-u.ac.jp](mailto:yoshimura.masahiko.8m@kyoto-u.ac.jp), [inose.tomoko.1v@kyoto-u.ac.jp](mailto:inose.tomoko.1v@kyoto-u.ac.jp)

**Table of Contents**

1. **Supplementary methods** S2

Chemicals and reagents

General reaction and purification and chemical analysis

Photochemical properity of coumarins and photoirradiation setup

Synthesis of small molecules

Analysis of photolysis of coumarins by HPLC

Analysis of hydrolysis of coumarins by TLC

Transient absorption measurements of coumarin derivatives

Protein preparation

Conjugation of photolinkers with HaloTag-NanoLuc

Protein release experiments

1. **Supplementary figures** S13

^1^H and ^13^C NMR of synthetic molecules

UV-vis and fluorescence spectra of coumarin derivatives

TLC analysis of hydrolysis of coumarins

Enzymatic hydrolysis resistance of compound **3** and **5**

HPLC analysis of photolysis of coumarins coumarin

Irradiation setup for the photocleavage of coumarin derivatives

Sructural analysis of photolytic product **6**

Plausible mechanism of desilylation upon photocleavage

Gel electrophoresis of HaloTag conjugated

1. **Supplementary table** S35
2. **Abbreviation** S36
3. **Supplementary references** S37
4. **Supplementary methods**
   1. **Chemicals and reagents**

All chemical reagents were purchased at the highest available commercial quality from BroadPharm, TCI, Sigma-Aldrich and BLDpharm, and used without further purification.

- 1. **General reaction, purification, and chemical analysis**

All reactions were carried out under an argon atmosphere with dry solvents unless otherwise noted. Chromatographic purifications were done on an Biotage Isolera^TM^ One equipped with standard silica columns (SepaFlash^TM^ SilicaFlash Cartridge UltraPure irregular Silica Gel, 40–63 μm, 60 Å from Santai Sicence Inc.). Thin-layer chromatography was performed on precoated TLC glass plates silica gel 60 matrix with fluorescence indicator UV_254_. The TLC plates were stained with iodine. ^1^H and ^13^C NMR spectra were recorded on a Bruker spectrometer operating at 500 and 126 MHz, respectively. Chemical shifts are given in ppm and calibrated using residual undeuterated solvent as an internal reference (In CDCl_3_, δ = 7.26 for ^1^H NMR and δ = 77.1 for ^13^C NMR).The following abbreviations describe the multiplets: s, singlet; d, doublet; dd, doublet of doublets; ddd, doublet of doublet of doublets; tdd, triplet of doublet of doublets; t, triplet; dt, doublet of triplets; q, quadruplet; tq, triplet of quadruplets; quin, quintet; sext. sextet; m, multiplet; br, broad. HRMS spectra were measured by ESI-orbitrap-MS: Exactive Plus or APCI-orbitrap-MS: Exactive Plus (Thermo Fisher Scientific).

- 1. **Photochemical properity of coumarins and photoirradiation setup**

UV-vis absorption spectra were measured by JASCO V-670 spectrophotometer. Fluorescence spectra were obtained with a HITACHI F-7000 fluorescence spectrometer. For the photocleavage experiment, LED light source (Asahi Spectra CL-1503 LED controller and 405 nm LED head) was used. In a time-dependent ^1^H NMR analysis upon photoirradiation, a stirred 1 mL solution of a coumarin compound (~10 mM) in *d^6^*-DMSO containing 1 % D_2_O was irradiated from the side of a quartz vial (optical path = 1 cm), using the LED-system. After irradiating for a specific duration, each sample was analyzed by ^1^H NMR to monitor changes in the spectra.

- 1. **Synthesis of small molecules**

Scheme S1. Synthesis scheme of coumarin with different substituents

Compound **10**, **12**, and **13** were synthesized accoding to previously reported procedures.^31, 45^

Synthesis of compound **2** (Carb **2**)

To a solution of compound **10** (10.0 mg, 0.04 mmol) in dichloroethane (0.4 mL, 0.1 M) was added *N*, *N*-diisopropylethylamine (DIPEA) (7.05 μL, 1.0 equiv). The mixture was stirred at RT for 5 minutes. Benzyl isocyanate (10 μL, 2.0 equiv.) and DMAP (2.8 mg, 0.6 equiv.) was added to the reaction solution at RT and stirred for 2.5 h. The reaction mixture was then diluted with DCM and washed twice with brine. The combined organic layer was dried over anhydrous MgSO_4_, filtrated, and concentrated under reduced pressure. The crude was purified by chromatography over silica gel with EtOAc and Toluene (1:4) to afford the desired carbamate **2** (12 mg, 79% yield) as yellow solid. ^1^H NMR (500 MHz, CDCl_3_) δ (ppm):1.21 (*t*, 6H, 7.0 Hz), 3.42 (*q*, 4H, 7.0 Hz), 4.42 (*d*, 2H, *^3^J* = 6.0 Hz), 5.26 (*s*, 2H), 5.33 (*br*, 1H), 6.18 (*s*, 1H), 6.63 (*br*, 1H), 6.73 (*br*, 1H), 7.27-7.37 *(m*, 6H). ^13^C NMR (126 MHz) δ (ppm): 12.3, 45.4, 46.0, 62.0, 107.4, 124.7, 127.6, 127.7, 127.8, 128.8, 128.9, 138.1, 150.2, 155.6, 156.1, 161.7. HRMS (APCI, Positive-mode): m/z calcd for [C_22_H_24_N_2_O_4_^+^H]^+^: 381.1809, found 381.1806.

Synthesis of compound **11**

To a solution of 7-(diethylamino)-2-oxo-2H-chromene-4-carbaldehyde (92.8 mg, 0.38 mmol) in dry THF (10 mL, 0.04 M) at –78 °C, ((trimethylsilyl)methyl) magnesium chloride in THF solution(1 mL, 3.0 equiv.) was added under an argon atmosphere. The mixture was stirred at – 78 °C to RT overnight, and subsequently, saturated qaueous NH_4_Cl was added. The reaction mixture was extracted twice with EtOAc. The combined organic layers were dried over anhydrous MgSO_4_, filtrated, and concentrated under reduced pressure. The crude was purified by chromatography over silica gel with EtOAc and toluene (1:4) to afford the desired carbamate **12** (53.6 mg, 42% yield) as yellow solid. ^1^H NMR (500 MHz, CDCl_3_) δ (ppm): 0.00 (*s*, 9H), 1.03 (*m*, 2H), 1.08 (*t*, 6H, *^3^J* = 7.2 Hz), 3.28 (*q*, 4H, *^3^J* = 7.2 Hz), 4.97 (*m*, 1H), 6.12 (*s*, 1H), 6.42 (*br*, 1H), 6.52 (*br*, 1H), 7.34 (*d*, 1H, 3J = 9.0 Hz) ^13^C NMR (126 MHz) δ (ppm): 0.7, 12.4, 26.6, 45.6, 68.5, 99.3, 105.0, 107.4, 109.6, 125.5, 149.5, 156.0, 161.0, 162.8. HRMS (APCI, Positive-mode): m/z calcd for [C_18_H_27_N_1_O_3_Si_1_^+^H]^+^: 334.1833, found 334.1827.

Synthesis of compound **3** (Si-Carb **3**)

To a solution of compound **11** (39.0 mg, 0.12 mmol) in toluene (4.0 mL, 0.03 M) was added *N*, *N*-diisopropylethylamine (DIPEA) (20 μL, 1.0 equiv). The mixture was stirred at RT for 5 minutes. Benzyl isocyanate (28.0 μL, 2.0 equiv.) was added to the reaction solution at RT and stirred overnight at 115 °C. The reaction mixture was diluted with EtOAc and washed twice with brine. The combined organic layer was dried over anhydrous MgSO_4_, filtrated and concentrated under reduced pressure. The crude was purified by chromatography over silica gel with EtOAc and Toluene (3:17) to afford the desired carbamate **3** (32.3 mg, 73% yield) as yellow solid. ^1^H NMR (500 MHz, CDCl_3_) δ (ppm): 0.08 (*s*, 9H), 1.17 - 1.26 (*m*, 8H), 3.28 (*q*, 4H, *^3^J* = 7.1 Hz), 4.36 (*m*, 2H), 5.23 (*br*, 1H), 6.03 (*t*, 1H, 7.4 Hz), 6.11 (*s*, 1H), 6.57 (*br*, 1H), 6.67 (*br*, 1H), 7.25 - 7.37 (*m*, 5H), 7.44 (*d*, 1H, *^3^J* = 9.1 Hz). ^13^C NMR (126 MHz) δ (ppm): - 0.9, 12.5, 24.6, 45.3, 45.4, 70.2, 99.1, 104.9, 109.6, 125.2, 127.4, 127.6, 127.7, 128.9, 138.3, 150.0, 155.3, 156.7, 157.8, 162.4. HRMS (APCI, Positive-mode): m/z calcd for [C_26_H_34_N_2_O_4_Si_1_^+^H]^+^: 467.2361, found 467.2359.

Synthesis of compound **4**

To a solution of compound **12** (55.0 mg, 0.21 mmol) in *p*-xylene (4.0 mL, 0.06 M) was added *N*, *N*-diisopropylethylamine (DIPEA) (36.5 μL, 1.0 equiv). The mixture was stirred at RT for 5 minutes. Benzyl isocyanate (52.0 μL, 2.0 equiv.) was added to the reaction solution at RT ands stirred overnight at 115 °C. The reaction mixture was diluted with EtOAc and washed twice with brine. The combined organic layer was dried over anhydrous MgSO_4_, filtrated, and concentrated under reduced pressure. The crude was purified by chromatography over silica gel with EtOAc and Toluene (1:4) to afford the desired carbamate **4** (61 mg, 73% yield) as yellow solid. ^1^H NMR (500 MHz, CDCl_3_) δ (ppm): 1.21 (*t*, 6H, 7.1 Hz), 1.57 (*d*, 3H, *^3^J* = 6.7 Hz), 3.41 (*q*, 4H, *^3^J* = 7.1 Hz), 4.33-4.42 (*m*, 2H), 5.23 (*t*, 1H, *^3^J* = 5.4 Hz), 6.04 (*q*, 1H, *^3^J* = 6.7 Hz), 6.15 (*s*, 1H), 6.52 (*d*, 1H, *^4^J* = 2.3 Hz), 6.61 (*d*, 1H, *^3^J* = 8.9 Hz), 7.28-7.36 *(m*, 5H), 7.42 (*d*, 1H, *^3^J* = 8.9Hz). ^13^C NMR (126 MHz) δ (ppm): 12.6, 21.2, 45.0, 45.4, 68.0, 98.4, 105.0, 106.2, 109.0, 125.0, 127.7, 127.8, 128.9, 138.2, 150.6, 155.4, 156.3, 156.7, 162.4. HRMS (APCI, Positive-mode): m/z calcd for [C_23_H_26_N_2_O_4_^+^H]^+^: 395.1965, found 395.1963.

Synthesis of compound **5**

To a solution of compound **13** (54.3 mg, 0.18 mmol) in dichloroethane (2.5 mL, 0.07 M) was added *N*, *N*-diisopropylethylamine (DIPEA) (32.0 μL, 1.0 equiv). The mixture was stirred at RT for 5 minutes. Benzyl isocyanate (44.0 μL, 2.0 equiv.) was added to the reaction solution at RT and stirred overnight at 85 °C. The reaction mixture was diluted with DCM and washed twice with brine. The combined organic layer was dried over anhydrous MgSO_4_, filtrated and concentrated under reduced pressure. The crude was purified by chromatography over silica gel with EtOAc and Toluene (1:4) to afford the desired carbamate **5** (68.1 mg, 87% yield) as yellow solid. ^1^H NMR (500 MHz, CDCl_3_) δ (ppm): 1.19 (*t*, 6H, 7.0 Hz), 1.77 (*s*, 3H), 1.94 (*s*, 3H), 3.40 (*q*, 4H, *^3^J* = 7.0 Hz), 4.37 (*d*, 2H, *^3^J* = 6.0 Hz), 5.20 (*t*, 1H, *^3^J* = 5.4 Hz), 5.27 (*d*, 1H, *^3^J* = 9.2 Hz), 6.17 (*s*, 1H), 6.49 (*d*, 1H, *^4^J* = 2.5 Hz), 6.55 (*d*, 1H, *^3^J* = 9.1 Hz), 6.59 (*d*, 1H, *^3^J* = 9.2 Hz) 7.26 - 7.39 *(m*, 5H). ^13^C NMR (126 MHz) δ (ppm): 12.5, 18.8, 25.8, 44.7, 45.3, 69.1, 98.0, 105.6, 106.3, 108.6, 121.4, 125.4, 127.5, 127.6, 128.7, 138.1, 140.7, 150.4, 154.7, 155.3, 156.6, 162.3. HRMS (APCI, Positive-mode): m/z calcd for [C_26_H_30_N_2_O_4_^+^H]^+^: 435.2278, found 435.2277.

Scheme S2. Synthesis scheme of azide-coumarin-Halotag linker

Compound **15** was synthesized according to a previously reported procedure.^46^

Synthesis of compound **16**

Under an Ar atmosphere, a 50 mL two-neck flask was charged with Compound **15** (521 mg, 1.69 mmol), *tert*-butyl piperazine-1-caboxylate (759 mg, 2.4 eqiv.), Pd_2_(dba)_3_･CHCl_3_ (34.7 mg, 0.02 equiv.), X-Phos (318 mg, 0.39 equiv.), Cs_2_CO_3_ (1.60 g, 2.91 equiv.) and dioxane (11 mL, 0.15 M). The mixture was refluxed overnight. The reaction mixture was filtrated through Celite and concentrated. The residue dissolved in EtOAc and washed twice with brine. The combined organic layers were dried over anhydrous MgSO_4_, filtrated, and concentrated under reduced pressure. The crude was purified by chromatography over silica gel (hexane : EtOAc 20 ~ 40 %) to afford the desired product **3** (343 mg, 59 % yield) as whitish yellow solid. ^1^H NMR (500 MHz, CDCl_3_) δ (ppm): 1.48 (*s*, 9H), 2.36 (*d*, 3H, *^4^J* = 1.2 Hz), 3.30 (*t*, 4H, *^3^J* = 5.1 Hz), 3.59 (*t*, 4H, *^3^J* = 5.1 Hz), 6.06 (*s*, 1H), 6.71 (*d*, 1H, *^4^J* = 2.5 Hz), 6.81 (*dd*, 1H, *^3^J* = 8.9 Hz, *^4^J* = 2.5 Hz), 7.44 (*d*, 1H, *^3^J* = 8.9 Hz). ^13^C NMR (126 MHz) δ (ppm): 18.6, 28.5, 43.3, 47.8, 80.4, 101.8, 111.2, 111.8, 112.1, 125.5, 152.6, 153.5, 155.5, 161.8. HRMS (ESI, Positive-mode): m/z calcd for [C_19_H_24_N_2_O_4_^+^Na]^+^: 367.1628, found 367.1632.

Synthesis of compound **17**

To solution of compound **16** (343 mg, 1.0 mmol) in p-xylene (10 mL, 0.1 M) was added selenium dioxide (212 mg, 1.92 equiv.) at 105 °C. The mixture was refluxed overnight. The reaction mixture was filtrated through Celite and concentrated. The residue dissolved with EtOAc and washed twice with brine. The combined organic layers were dried over anhydrous MgSO_4_, filtrated and concentrated under reduced pressure. The crude was purified by chromatography over silica gel (toluene : EtOAc = 17 : 3) to afford the desired product **17** (158 mg, 44% yield) as red solid. ^1^H NMR (500 MHz, CDCl_3_) δ (ppm): 1.48 (*s*, 9H), 3.36 (*t*, 4H, *^3^J* = 5.5 Hz), 3.60 (*t*, 4H, *^3^J* = 5.5 Hz), 6.59 (*s*, 1H), 6.71 (*d*, 1H, *^4^J* = 2.6 Hz), 6.84 (*dd*, 1H, *^3^J* = 9.2 Hz, *^4^J* = 2.5 Hz), 8.39 (*d*, 1H, *^3^J* = 9.2 Hz) 10.0 (*s*, 1H). ^13^C NMR (126 MHz) δ (ppm): 28.5, 43.1, 47.2, 80.5, 101.1, 106.3, 112.2, 120.3, 127.2, 134.8, 153.7, 154.7, 156.9, 161.4, 192.2. HRMS (ESI, Positive-mode): m/z calcd for [C_19_H_22_N_2_O_5_+Na]^+^: 381.1421, found 381.1426.

Synthesis of compound **18**

To solution of compound **18** (70.0 mg, 0.2 mmol) in methanol (10 mL, 0.02 M) NaBH_4_ (14.4 mg, 1.95 equiv.) was added. The mixture was stirred for 20 minutes, and subsequently, aqueous HCl was added. The reaction mixture was extracted twice with DCM. The combined organic layers were dried over anhydrous MgSO_4_, filtrated, and concentrated under reduced pressure. The crude was purified by chromatography over silica gel (DCM : MeOH 0~5%) to afford the desired product **18** (42 mg, 59% yield) as whitish yellow solid. ^1^H NMR (500 MHz, CDCl_3_) δ (ppm):1.48 (*s*, 9H), 3.20 (*t*, 4H, *^3^J* = 5.5 Hz), 3.59 (*t*, 4H, *^3^J* = 5.5 Hz), 4.85 (*d*, 2H, *^4^J* = 1.3 Hz), 6.38 (*t*, 1H, *^4^J* = 1.3 Hz), 6.71 (*d*, 1H, *^4^J* = 2.4 Hz), 6.71 (*dd*, 1H, *^3^J* = 9.0 Hz, *^4^J* = 2.4 Hz), 7.38 (*d*, 1H, *^3^J* = 9.0 Hz). ^13^C NMR (126 MHz) δ (ppm): 28.5, 43.5, 47.7, 61.0, 80.5, 101.9, 108.0, 109.4, 111.8, 124.4, 153.4, 154.4, 154.8, 155.7, 162.1. HRMS (ESI, Positive-mode): m/z calcd for [C_19_H_24_N_2_O_5_^+^Na]^+^: 383.1577, found 383.1580.

Synthesis of compound **19**

Under an argon atmosphere, a solution of compound **18** (29.2 mg, 0.08 mmol) in dry MeOH (2 mL, 0.04 M) was added to TMSCl (0.5 mL). The mixture was stirred for 30 minutes, then the solvent was removed by N_2_ flow. The resulting mixture (compound **18’**) was used in the next reaction without further purification. A solution of 4-azide benzoic acid (13.0 mg, 0.08 mmol) in DMF (0.8 mL, 0.1 M) was added to EDC (17.0 mg, 1.1 equiv.), HOBt (14.3 mg, 1.3 equiv.), and NMM (26.5 μL, 3.0 equiv.) at 0 °C. The mixture was stirred for 20 minutes, then compound **18’** in DMF solution (1.0 mL) was added. The reaction mixture was warmed to RT and stirred for two hours, then subsequently diluted with EtOAc. The organic layer was washed with 10 % LiCl aq, saturated NH_4_Cl aq, and saturated NaHCO_3_ aq. The combined organic layers were dried over anhydrous MgSO_4_, filtrated, and concentrated under reduced pressure to afford the desired compound **21** (39.7 mg, 83 % yield) as yellow solid. ^1^H NMR (500 MHz, CDCl_3_) δ (ppm): 3.36 (*br*, 4H), 3.75 (*br*, 4H), 4.84 (*d*, 2H, *^4^J* = 1.3 Hz ), 6.39 (*t*, 1H, *^4^J* = 1.3 Hz), 6.73 (*d*, 1H, *^4^J* = 2.5 Hz), 6.81 (*dd*, 1H, *^3^J* = 9.0 Hz, *^4^J* = 2.5 Hz), 7.08 (*pseudo-d*, 2H), 7.39 (*d*, 1H, *^3^J* = 9.0 Hz), 7.46 (*pseudo-d*, 2H). ^13^C NMR (126 MHz) δ (ppm): 48.0, 61.0, 102.2, 108.4, 109.8, 112.0, 119.3, 124.5, 129.3, 131.6, 142.3, 153.2, 154.2, 155.7, 161.9, 169.9. HRMS (ESI, Positive-mode): m/z calcd for [C_21_H_19_N_5_O_4_^+^Na]^+^: 428.1329, found 428.1335.

Synthesis of compound **20**

Under an argon atmosphere, to a solution of compound **17** (225.7 mg, 0.63 mmol) in dry THF (10 mL, 0.06 M) at –78 °C, ((trimethylsilyl)methyl) magnesium chloride in THF solution (1 mL, 1.6 equiv.) was added. The mixture was stirred from – 78 °C to RT overnight, and subsequently, saturated aqueous NH_4_Cl was added. The reaction mixture was extracted twice with DCM. The combined organic layers were dried over anhydrous MgSO_4_, filtrated, and concentrated under reduced pressure. The crude was purified by chromatography over silica gel with EtOAc and Toluene (1 : 4) to afford the desired carbamate **20** (153.3 mg, 55% yield) as yellow solid. ^1^H NMR (500 MHz, CDCl_3_) δ (ppm): 0.10 (*s*, 9H), 1.12 (*m*, 2H), 1.46 (*s*, 9H), 3.27 (*t*, 4H, *^3^J* = 5.5 Hz), 3.56 (*t*, 4H, *^3^J* = 5.5 Hz), 5.07 (*m*, 1H), 6.30 (*s*, 1H), 6.64 (*br*, 1H), 6.77 (*d*, 1H, *^3^J* = 8.7 Hz), 7.50 (*d*, 1H, *^3^J* = 8.7 Hz). ^13^C NMR (126 MHz) δ (ppm): - 0.7, 26.4, 28.5, 43.0, 47.6, 68.4, 80.4, 101.9, 106.5, 109.1, 111.6, 125.3, 153.0, 154.7, 156.0, 160.9, 162.5. HRMS (ESI, Positive-mode): m/z calcd for [C_23_H_34_N_2_O_5_Si^+^Na]^+^:469.2129, found 469.2138.

Synthesis of compound **21**

Under an argon atmosphere, a solution of compound **20** (43.0 mg, 0.1 mmol) in dry MeOH (2 mL, 0.05 M) was added to TMSCl (0.5 mL). The mixture was stirred for two hours, then the solvent was removed by N_2_ flow. The resulting mixture (compound **20’**) was used in the next reaction without further purification. A solution of 4-azide benzoic acid (20.2 mg, 0.12 mmol) in DMF (1 mL, 0.12 M) was added to EDC (34.5 mg, 1.9 equiv.), HOBt (23.8 mg, 1.8 equiv.), NMM (42 µL, 4.0 equiv.) at 0 °C. The mixture was stirred for 20 minutes, then compound **21’** in DMF solution (1.0 mL) was added. The reaction mixture was warmed to RT and stirred for two hours, subsequently diluted with EtOAc. The organic layer was washed with 10 % LiCl aq, saturated NH_4_Cl aq, and saturated NaHCO_3_ aq. The combined organic layers were dried over anhydrous MgSO_4_, filtrated, and concentrated under reduced pressure to afford the desired compound **21** (39.7 mg, 83% yield) as yellow solid. ^1^H NMR (500 MHz, CDCl_3_) δ (ppm): 0.11(s, 9H), 1.14 (m, 2H), 3.35 (*br*, 4H), 3.78 (*br*, 4H), 5.06 (*m*, 1H, ), 6.32 (*s*, 1H,), 6.70 (*s*, 1H), 6.80 (*d*, 1H, *^3^J* = 8.8 Hz), 7.06 (*pseudo-d*, 2H), 7.44 (*pseudo-d*, 2H), 7.44 (*d*, 1H, *^3^J* = 8.8 Hz). ^13^C NMR (126 MHz) δ (ppm): - 0.7, 26.5, 47.9, 68.4, 102.2, 106.9, 109.5, 111.8, 119.3, 125.4, 129.3, 131.6, 142.3, 152.8, 156.1, 160.7, 162.3, 169.9. HRMS (ESI, Positive-mode): m/z calcd for [C_25_H_29_N_5_O_4_Si^+^Na]^+^: 514.1881, found 514.1890.

Synthesis of compound **7**

To a solution of HaloTagLigand amine HCl salt (26.0 mg, 0.1 mmol) in dichloromethane (1.0 mL, 0.1 M), succinic anhydride (10.0 mg, 1.0 equiv.) and NEt_3_ (26 μL, 2.0 equiv.) were added. After stirring at RT for 30 min, the solvent was removed in vacuo. The resulting residue was dissolved in 2 M HCl aqueous solution, then the desired molecule was extracted from EtOAc 3 times. The combined organic phase was dried over MgSO_4_, filtrated and concentrated to obtain the condensate of the amine and succinic anhydride (**22)**. The crude material was used for the next reaction without any purification.

To a solution of compound **19** (22.7 mg, 0.06 mmol), EDC (37.3 mg, 3.5 equiv.) and DMAP (1.0 mg, 0.15 equiv.) in dichlroethane (1.0 mL, 0.06 M) was added a solution of **22** (23.5 mg, 1.3 equiv) in acetonitrile (1 mL). The reaction was stirred for 1.5 hours. The reaction mixture was diluted with EtOAc and washed with brine twice. The combined organic phase was dried over anhydrous MgSO_4_, filtrated, and concentrated under reduced pressure. The crude was purified by PTLC (EtOAc) to afford the desired carbamate **7** (19.2 mg, 55 % yield) as a yellow solid. ^1^H NMR (500 MHz, CDCl_3_) δ = 1.28 - 1.42 (*m*, 4H), 1.50 - 1.57 (*m*, 2H), 1.68 - 1.73 (*m*, 2H), 2.48 (*t*, 2H, *^3^J* = 6.8 Hz), 2.73 (*t*, 2H, *^3^J* = 6.8 Hz), 3.22 - 3.88 (*m*, 20H), 5.18 (*d*, 2H, *^4^J* = 1.2 Hz), 6.06 (*br*, 1H), 6.20 (*s*, 1H), 6.66 (*d*, 1H, *^4^J* = 2.5 Hz), 6.75 (*dd*, 1H, *^3^J* = 8.9 Hz, *^4^J* = 2.5 Hz), 7.02 (*pseudo-d*, 2H), 7.31 (*d*, 1H, *^3^J* = 8.9 Hz), 7.40 (*pseudo-d*, 2H). ^13^C NMR (126 MHz) δ (ppm): 25.6, 26.8, 29.6, 32.7, 41.2, 45.2, 47.9, 61.7, 69.9, 70.2, 70.5, 71.5, 102.1, 108.9, 109.4, 112.0, 119.3, 124.5, 129.3, 131.7, 142.3, 150.2, 153.3, 155.6, 155.7, 161.4, 169.9. HRMS (ESI, Positive-mode): m/z calcd for [C_21_H_19_N_5_O_4_^+^Na]^+^:733.2737, found 733.2723.

Synthesis of compound **8**

To a solution of compound **19** (12.6 mg, 0.03 mmol) and N,N'-Disuccinimidyl carbonate (11.5 mg, 1.4 equiv.) in dichlroethane and acetonitrile (1 : 1) (1.0 mL, 0.03 M) was added DMAP (5.5 mg, 1.5 equiv). The reaction was monitored by TLC until compound **19** was fully consumed. HaloTag ligand amine HCl salt (12.6 mg, 1.6 equiv.) was then added to the reaction solution at RT, and the solution was stirred for 20 minutes. The reaction mixture was diluted with EtOAc and washed with brine twice. The combined organic phase was dried over anhydrous MgSO_4_, filtrated, and concentrated under reduced pressure. The crude was purified by PTLC (toluene : EtOAc = 1 : 1) to afford the desired carbamate **8** (9.2 mg, 45% yield) as yellow solid. ^1^H NMR (500 MHz, CDCl_3_) δ = 1.28 - 1.42 (*m*, 4H), 1.51 - 1.57 (*m*, 2H), 1.66 - 1.72 (*m*, 2H), 3.22 - 3.83 (*m*, 20H), 5.18 (*s*, 2H), 5.45 (*br*, 1H), 6.19 (*s*, 1H), 6.60 (*d*, 1H, ^4^*J* = 2.5 Hz), 6.75 (*dd,* 1H, ^3^*J* = 9.0 Hz, ^4^*J* = 2.5 Hz), 7.02 (*pseudo-d*, 2H), 7.31 (*d*, ^3^*J* = 9.0 Hz), 7.40 (*pseudo-d*, 2H) . ^13^C NMR (126 MHz) δ (ppm): 25.6, 26.8, 29.6, 32.7, 41.2, 45.2, 47.9, 61.7, 69.9, 70.2, 70.5, 71.5, 102.1, 108.9, 109.4, 112.0, 119.3, 124.5, 129.3, 131.7, 142.3, 150.2, 153.3, 155.6, 155.7, 161.4, 169.9. HRMS (ESI, Positive-mode): m/z calcd for [C_32_H_39_ClN_6_O_7_^+^Na]^+^:677.2461, found 677.2472.

Synthesis of compound **9**

To a solution of compound **21** (67.8 mg, 0.14 mmol) in acetonitrile (1.4 mL, 0.1 M) was added *N*, *N*-diisopropylethylamine (DIPEA) (72 μL, 3.0 equiv). The mixture was stirred at RT for 5 minutes, then N,N'-disuccinimidyl carbonate (75.8 mg, 2.0 equiv.) and DMAP (2.7 mg, 0.16 equiv.) were added. The reaction was monitored by TLC until compound **21** was fully consumed. HaloTag ligand amine HCl salt (69 mg, 0.27 mmol, 1.6 equiv.) was added to the reaction solution at RT, and the solution was stirred for 1.5 hours. The reaction mixture was diluted with EtOAc and washed with brine three times. The combined organic phase was dried over anhydrous MgSO_4_, filtrated, and concentrated under reduced pressure. The crude was purified by chromatography over silica gel (CHCl_3_:MeOH 0-3%) and PTLC (toluene: EtOAc = 7:3) to afford the desired carbamate **9** (20.2 mg, 20 % yield) as yellow solid. ^1^H NMR (500 MHz, CDCl_3_) δ (ppm): 0.07 (*s*, 9H), 1.21 (*d*, 2H, *^3^J* = 7.5 Hz), 1.36 - 1.40 (*m*, 2H), 1.43 - 1.48 (*m*, 2H), 1.59 - 1.65 (*m*, 2H), 1.74 - 1.80 (*m*, 2H), 3.30 - 3.88 (*m*, 20H), 5.37 (*t*, 1H, *^3^J* = 5.4 Hz), 5.98 (*t*, 1H, *^3^J* = 7.5 Hz), 6.20 (*s*, 1H), 6.71 (*d*, 1H, ^4^J = 2.5 Hz), 6.83 (*dd*, 1H, *^3^J* = 9.0 Hz, *^4^J* = 2.5 Hz), 7.09 (*pseudo-d*, 2H), 7.46 - 7.49 (*m*, 3H). ^13^C NMR (126 MHz) δ (ppm): - 0.9, 24.5, 25.5, 26.8, 29.6, 32.7, 41.1, 45.2, 47.9, 69.8, 70.0, 70.2, 70.6, 71.4, 102.3, 106.9, 109.2, 111.9, 119.3, 125.1, 129.3, 131.7, 142.3, 153.1, 155.3, 156.1, 157.7, 161.9, 169.8. HRMS (ESI, Positive-mode): m/z calcd for [C_36_H_49_ClN_6_O_7_Si^+^Na]^+^:763.3013, found 763.3031.

- 1. **Analysis of photolysis of coumarins by HPLC**

***Photolytic analysis***

An aqueous solution of coumarin compounds **1-3** (2 µM, 0.1% DMSO) was prepared with MilliQ water. The prepared solution was placed into the quartz vial and irradiated with 405 nm (6.6 mW/cm^2^) light while stirring at room temperature for 15, 30, 60, and 120 seconds. After irradiation, the solution was filtered using a 0.45 µm syringe filter to remove any particulates before analysis. The filtered solution was subjected to quantitative HPLC-MS analysis.

***Quantitative HPLC-MS analysis***

HPLC-MS was performed using Mass Spectrometer LCMS-2020 from Shimadzu equipped with an electrospray source (Polarity: positive ions, Capillary voltage: 4.5 kV, DL voltage: 0.0 V, Q-array RF voltage: 10.1 V, Source Temperature: 350°C, Desolvation Temperature: 250°C, Cone Gas Flow: 1.5 L/min, Desolvation Gas Flow: 15 L/min, Mass range (m/z): scan 100 to 500, SIM 248 and 352 (for Ester **1**), 248 and 381 (for Carb **2**), 244, 334, and 467 (for Si-Carb **3**) and an Prominence UFLC from Shimadzu: Binary pump (LC-20AD), heated column compartment (CTO-20AC), diode-array detector (SPD-M20A). Column: CAPCELL PAK C18 MG, 5.0 μm, 4.6 x 250 mm, Temp: 30°C, DAD Wavelength range (nm): 200 to 600 (380 nm was used to calculate the amounts of substrate/products). Runtime: 20 min (detectors: 0-15 min); Solvents: A = water, B= acetonitrile; Flow (ml/min) 0.8, Gradient: 70-100% B in 4.0 min, 100% B isocratic for 11.0 min, 100-70% B in 1.0 min, 70% B isocratic for 4.0 min.

- 1. **Analysis of hydrolysis of coumarins by TLC**

***Hydrolytic analysis***

An aqueous solution of coumarin compounds **1-3** and **5** (10 µM, 0.1% DMSO) was prepared with 50 mM Tris-HCl and 100 mM NaCl. To investigate non-enzymatic conditions, we prepared three different pH solutions: 5.3, 7.3, and 9.1. To investigate enzymatic conditions, the commercially available solution containing esterase from porcine liver (≥150 units/mg protein (biuret), Sigma-Aldrich) was diluted five-fold with buffer (50 mM Tris-HCl, 100 mM NaCl, pH 7.5). The aqueous solutions containing coumarin compounds **1-3** and **5** were incubated at 37 °C in the dark, and the time-dependent hydrolysis was tracked by quantitative TLC analysis.

***Quantitative TLC analysis***

5 µL of the reaction solutions were taken and spotted on the TLC plate. After drying the residual moisture with a dryer, the plate was immersed in a developing chamber with CHCl_3_:MeOH 99:1 as the eluent. After the development of spots, coumarin-derived fluorescence was detected using the luminescence image analyzer (LAS-4000, Fujifilm) with an exposure light of 365 nm and a detection filter, 510DF10. The acquired images were analyzed by ImageJ software, and the gray value of each spot was plotted against time.

- 1. **Transient absorption measurements of coumarin derivatives**

***Transition absorption spectral measurements***

Transient absorption (TA) spectra were recorded to observe spectral changes induced by light excitation. Excitation was provided by the third harmonic of a Nd:YAG pulsed laser (355 nm, Amplitude, SureLite2) with a pulse energy of 1.0 mJ. A xenon lamp (Max-302, Asahi Spectra) served as the probe light. Spectra were detected with a charge-coupled device (CCD) camera coupled to a spectroscope. The gate width of the CCD camera was set to 100 µs, and the delay time between the Nd:YAG laser and the CCD camera trigger was fixed to 25 ms using a delay generator to obtain the spectra at a specific time point.

***Single-Wavelength Transient Absorption Measurements***

Transient absorption (TA) signals were measured at 385 nm, a wavelength where coumarin derivatives exhibit significant absorption changes. The same Nd:YAG laser and xenon lamp were used as the excitation and probe light sources, respectively. The transmitted probe light was detected using a photomultiplier tube with a wavelength-selectable monochromator, and the temporal evolution of the transmitted signal was recorded using an oscilloscope (Agilent, DSO9054H). The signals were expressed as the ratio of the change in absorbance to the original absorbance at the same wavelength (ΔA/A) and analyzed using a bi-exponential function:

$\Delta A=A_{1}\exp\left( -\frac{t}{\tau_{1}} \right)+A_{2}\exp\left( -\frac{t}{\tau_{2}} \right)+A_{plateau}$ eqn (S1)

where A_1_ and A_2_ represent the amplitudes of the fast and slow components, τ_1_ and τ_2_ are their respective decay time constants, and Aplateau corresponds to the long-lived component. To enhance the signal-to-noise ratio, several hundred signals were averaged.

***Sample Conditions for TA measurements***

Samples were dissolved in dimethyl sulfoxide (DMSO) and adjusted the concentration to achieve the same absorbance at 355 nm to equalize the number of photons absorbed between samples. A flow system was employed to provide fresh sample solution during the measurements. All experiments were conducted at room temperature.

- 1. **Protein preparation**

***Construction of a recombinant cDNA plasmid***

The DNA sequences of HaloTag and NanoLuc were retrieved from Promega (Catalog number: G6591), addgene (https://www.addgene.org/188319/), and the reference article (PMID: 26569370), respectively. The DNA sequences were synthesized by Eurofins Genomics. Overlapping sequences were added at the 5’ and 3’ ends for seamless cloning. For the construction, the experimental protocol followed the In-Fusion HD cloning Kit from Takara (Catalog number: 639650). For detailed information on the construct, see Supplementary Table 1.

***Preparation of recombinant His-tagged-HaloTag-NanoLuc***

The recombinant His-tagged-HaloTag-NanoLuc was prepared from BL21(DE3) *E.coli* strain. The bacteria were incubated with LB medium at 37 °C until at OD_600_ = 0.6, then added 1 mM of IPTG, and continuously cultured at 28 °C for 5 hours. After collection by centrifugation with 8,000 g for 20 min, the bacterial pellets were sonicated with a wash buffer (20 mM HEPES (pH 7.5), 500 mM NaCl, 50 mM MgCl_2_, 1 mM DTT, 10 mM imidazole). To remove the debris, the samples were centrifuged with 15,000 g for 30 min, then the supernatant were purified using Ni-NTA agarose resin (QIAGEN, #30210) following manufacturer protocol with customized buffer (Wash buffer: 20 mM HEPES (pH 7.5), 500 mM NaCl, 50 mM MgCl_2_, 1 mM DTT , 10 mM imidazole, Elution buffer: 20 mM HEPES (pH 7.5), 500 mM NaCl, 20 mM MgCl_2_, 1 mM DTT , 200 mM imidazole). The elution solution was further purified by gel filtration using HiLoad ^®︎^16/600 superdex ^®︎^ 75 pg column in an AKTA pure system (Flow rate: 1 mL/min, Running buffer: 20 mM HEPES (pH 7.5), 500 mM NaCl, 1 mM DTT). The purified protein solution was divided into small portions, and frozen in liquid nitrogen. The recombinant protein samples were stored at -80 ◦C until use.

- 1. **Conjugation of photolinkers (7-9) with HaloTag-NanoLuc**


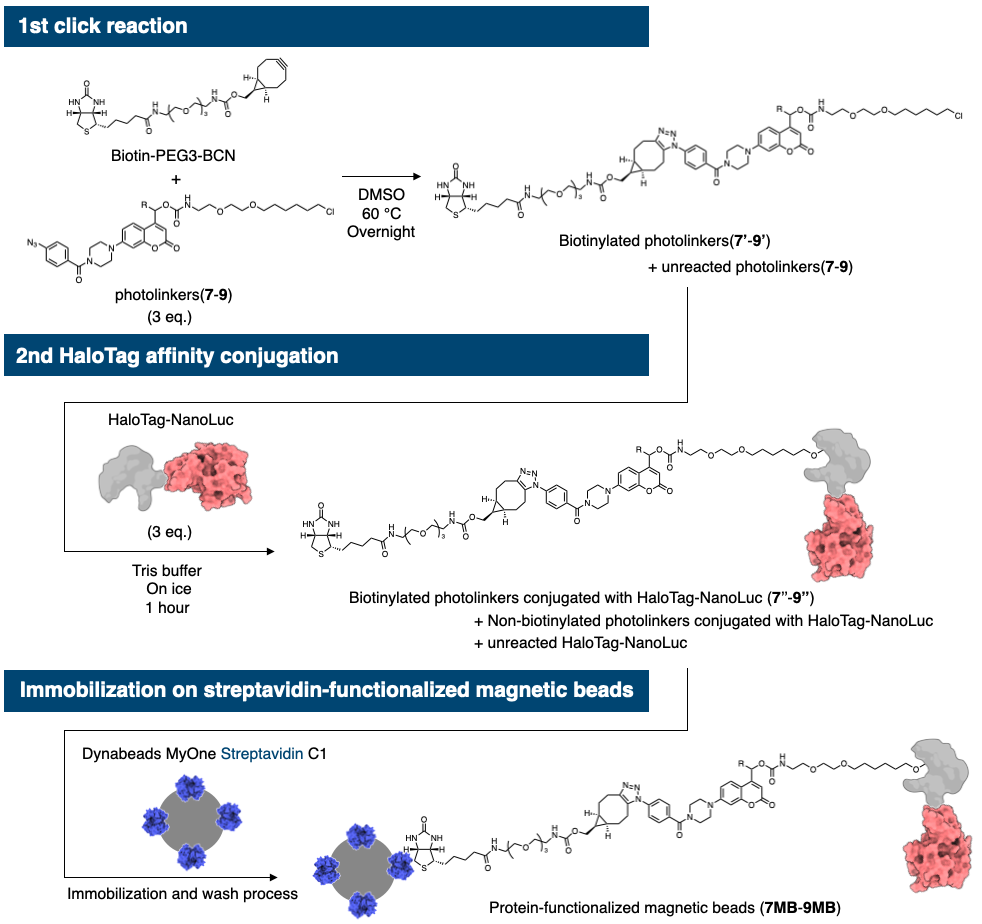


Scheme S3. Immobilization protocol of HaloTag-NanoLuc on the magnetic beads with photolinkers

To biotinylate the synthesized photolinkers (**7**-**9**), 15 μL of each coumarin compound (10 mM) in DMSO was mixed with 5 μL of BCN-PEG3-Biotin (10 mM) in DMSO and incubated at 60 ºC overnight (Scheme S3). After the overnight reaction, the full consumption of BCN-PEG3-Biotin was monitored by TLC with iodine staining, and the newly generated biotinylated compound was detected by coumarin-dependent fluorescence on TLC.

Subsequently, the obtained biotinylated photolinkers (**7’**-**9’**) were conjugated with HaloTag-NanoLuc. 1 μL of biotinylated photolinkers (**7’**-**9’**) (500 μM) and 100 μL of HaloTag-NanoLuc (15 μM) were mixed and incubated on ice for one hour. The final concentration of DMSO in the reaction solution was adjusted to less than 1 %. After the incubation, the full conversion of photolinkers (**7**-**9**) and biotinylated photolinkers (**7’**-**9’**) into the corresponding conjugats with HaloTag-NanoLuc was verified by sodium dodecyl sulfate polyacrylamide gel electrophoresis (SDS-PAGE) analysis.

Next, the desired biotinylated photolinkers conjugated with HaloTag-NanoLuc (**7”**-**9”**) were immobilized and purified on the streptavidine-functionalized magnetic beads (Dynabeads MyOne Streptavidin C1) using biotin-avidin interaction. The streptavidine-functionalized magnetic beads were washed twice with a buffer solution (50 mM Tris-HCl, 100 mM NaCl, pH 7.5) before use. Then, 30 µL of magnetic beads suspension were mixed with 300 µL of solution containing coumarin coumpounds (**7**”-**9**”) (10 nM) and incubated on ice for one hour. After the immobilization, the beads were washed three times with buffer (50 mM Tris-HCl, 100 mM NaCl, pH 7.5) and resuspended in 3 mL of the same buffer for the further analysis.

**1-10. Protein release experiments**

For the photo-stimulated protein release experiment, 50 μL of suspension of protein (**7”**- **9”**)-functionalized magnetic beads (**7MB**-**9MB**) were placed in a 96 well plate (Low-binding surface (LBS) 1/2 Area OptiPlate, 96-well (white) from PerkinElmer). Each sample suspension was then irradiated with 405 nm light at 10.7 mW/cm^2^ for different time duration (from a few seconds to 180 seconds). After the irradiation, 30 μL of the supernatant was taken and mixed with 30 μL NanoLuc substrate solution (Nano-Glo^®^ Luciferase Assay System, Promega) at room temperature. One minute after adding the substrate, the luminescence intensity of each supernatant was measured by a plate reader (infinite F500, Tecan) at the following setting: room temperature, 200 ms of measurement time, to quantitatively analyze the protein release from nanoparticles by photo-stimulation.

To investigate the hydrolytic protein release, 3 mL of the protein (**7”**- **9”**)-functionalized magnetic beads (**7MB**-**9MB**) prepared above were redispersed in 3 mL of different pH buffers (50 mM Tris-HCl, 100 mM NaCl) at pH 5.3, 7.3, and 9.1, and incubated for 30 minutes at 37°C. After incubation, 30 μL of the supernatant was taken and mixed with 30 μL NanoLuc substrate solution at room temperature and the luminescence intensity was measured.

To investigate the protein release by enzymatic hydrolytisis, the commercially available solution containing esterase from porcine liver (≥150 units/mg protein (biuret), Sigma-Aldrich) was diluted five-fold with buffer (50 mM Tris-HCl, 100 mM NaCl, pH 7.5). 1 μL of the diluted esterase solution was mixed with 50 uL of protein (**7”**- **9”**)-functionalized magnetic beads (**7MB**-**9MB**) dispersion and incubated for 30 min. at 37 ºC. After incubation, 30 μL of the supernatant was taken and mixed with 30 μL NanoLuc substrate solution at room temperature and the luminescence intensity was measured.

1. **Supplementary figures**

**2-1. ^1^H and ^13^C NMR of synthetic small molecules**

^
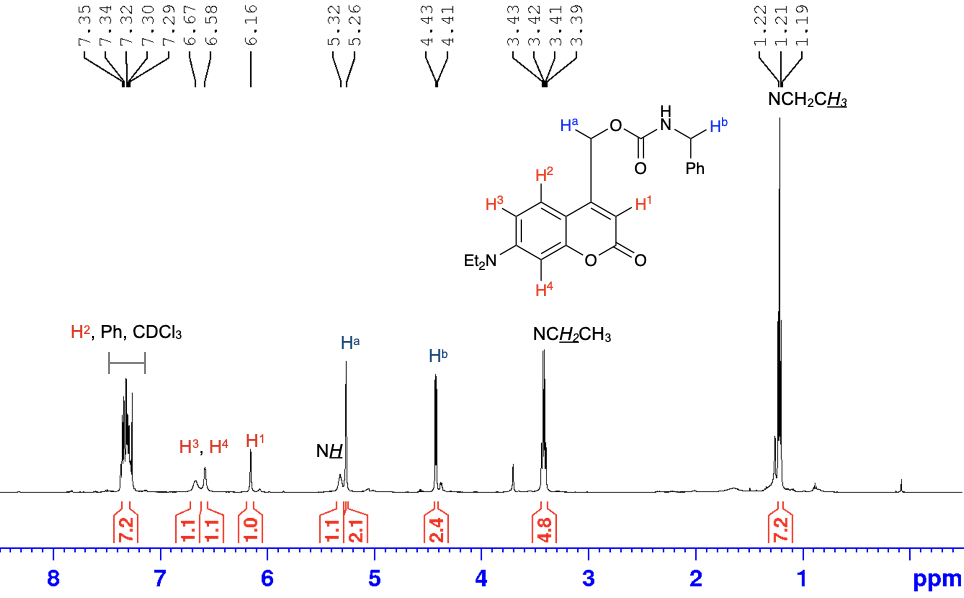
^

Figure S1. ^1^H NMR of compound **2**

^
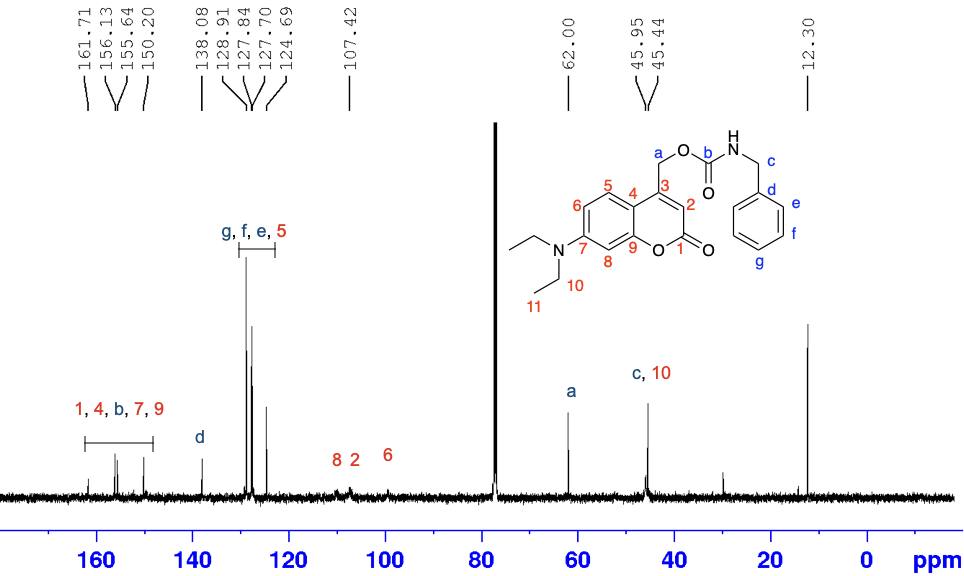
^

Figure S2. ^13^C NMR of compound **2**

^
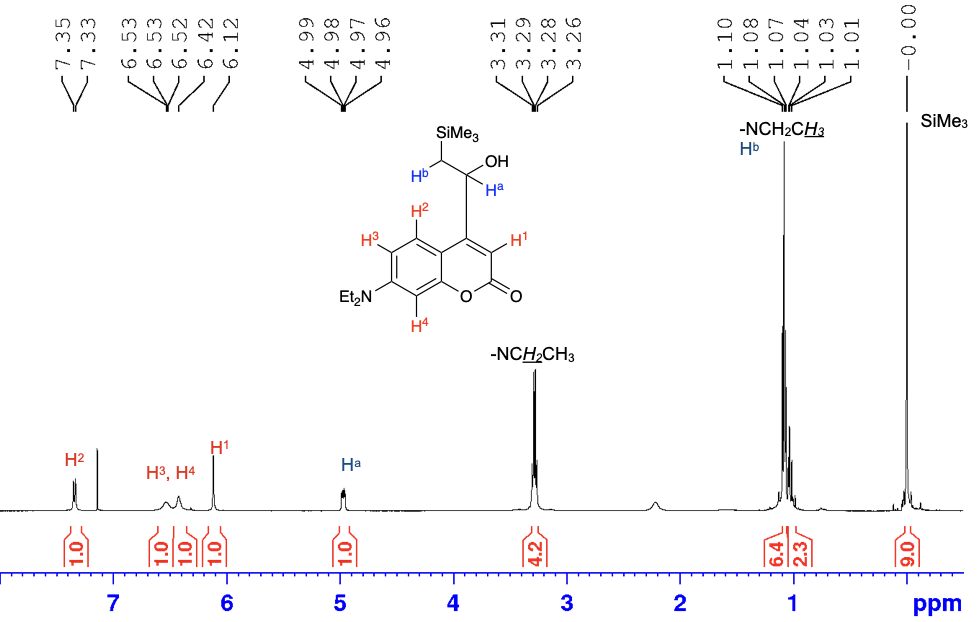
^

Figure S3. ^1^H NMR of compound **11**

^
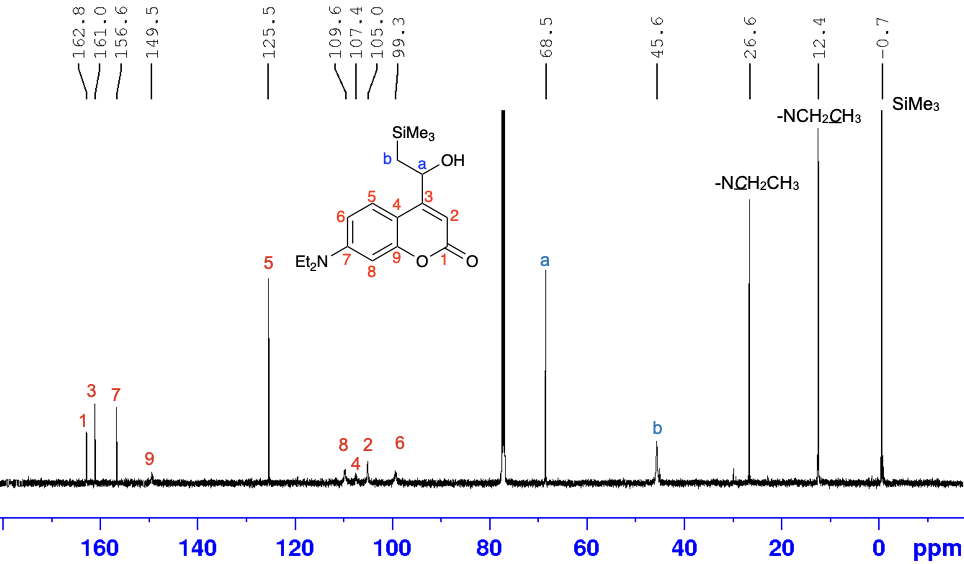
^Figure S4. ^13^C NMR of compound **11**

^
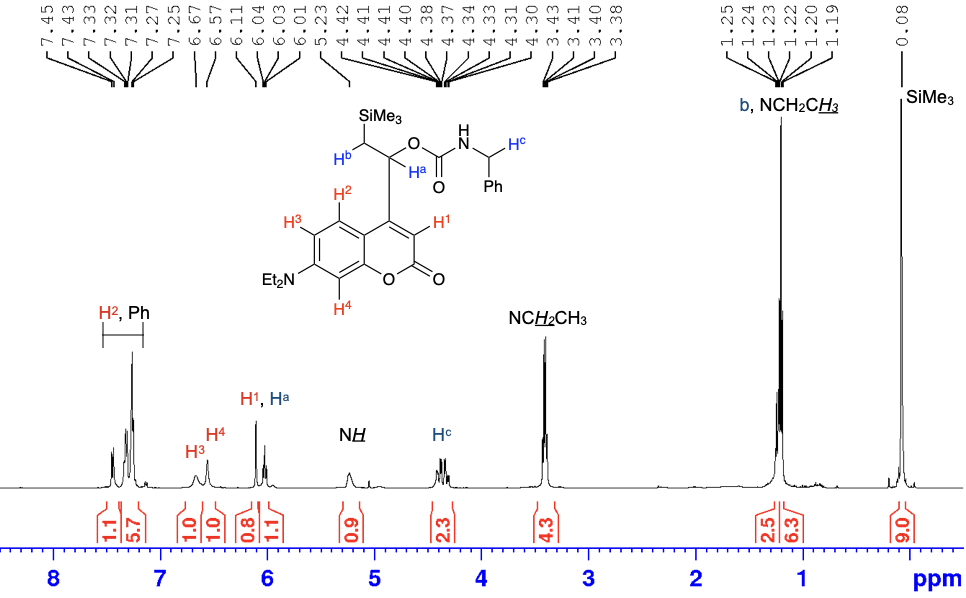
^

Figure S5. ^1^H NMR of compound **3**

^
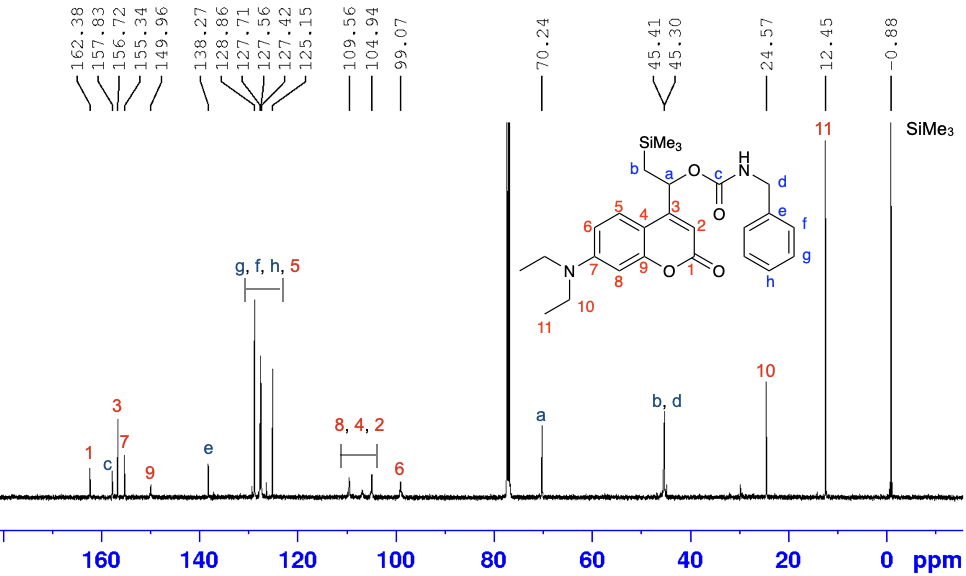
^

Figure S6. ^13^C NMR of compound **3**

^
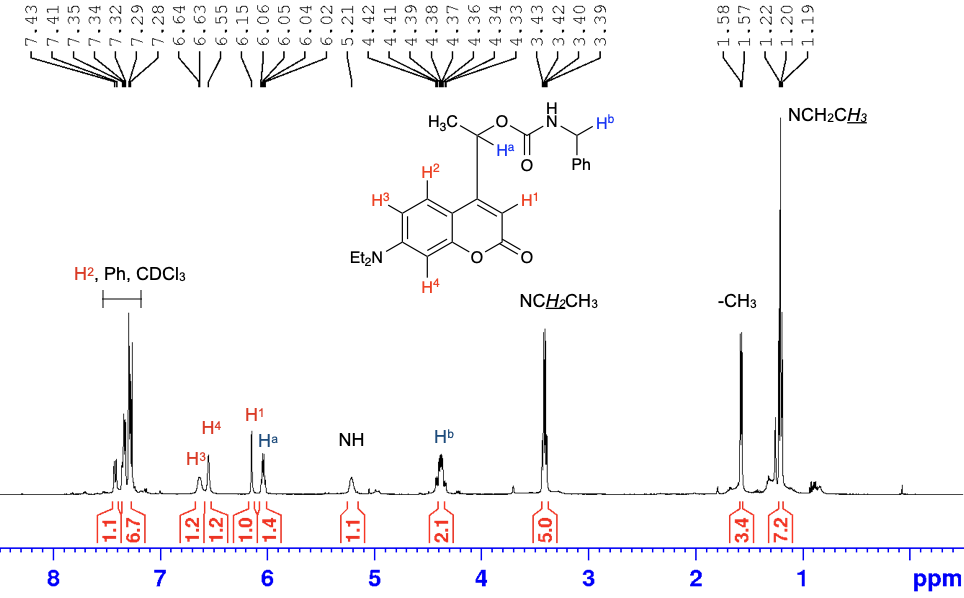
^

Figure S7. ^1^H NMR of compound **4**

^
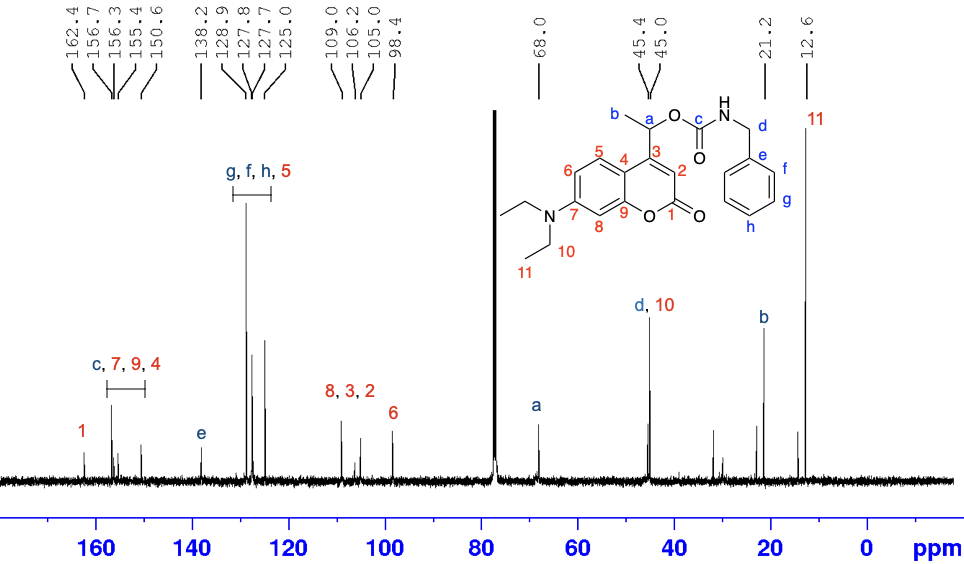
^

Figure S8. ^13^C NMR of compound **4**

^
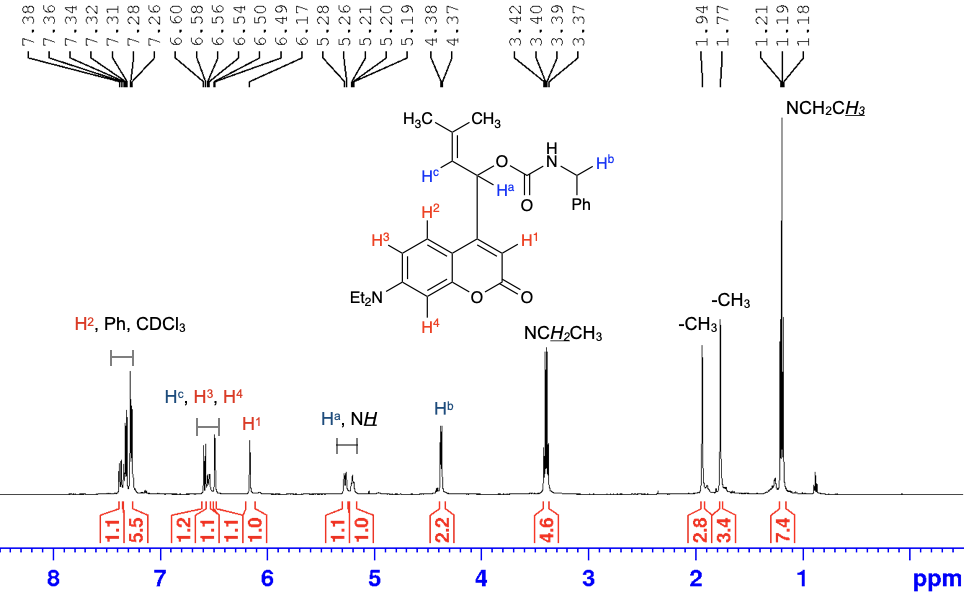
^

Figure S9. ^1^H NMR of compound **5**

^
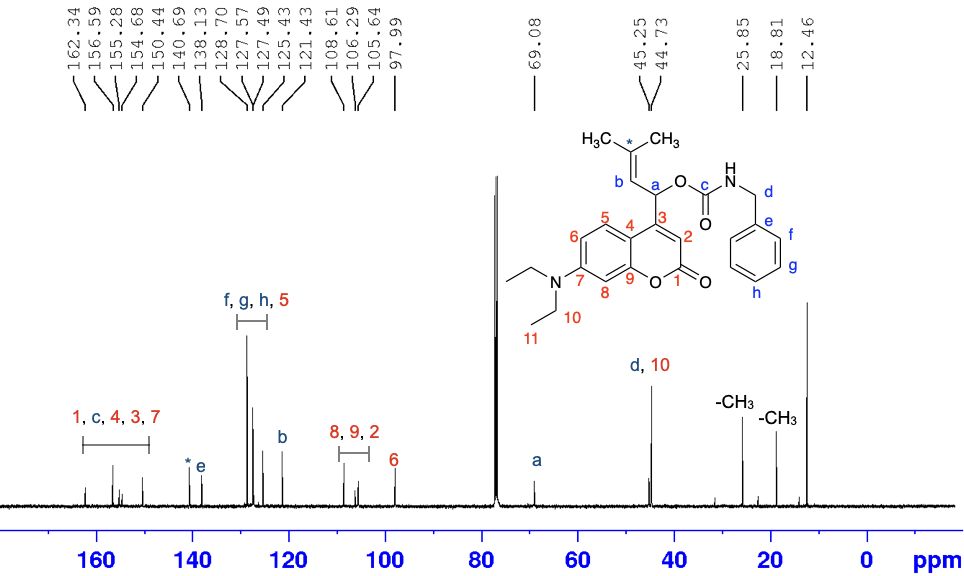
^

Figure S10. ^13^C NMR of compound **5**

^
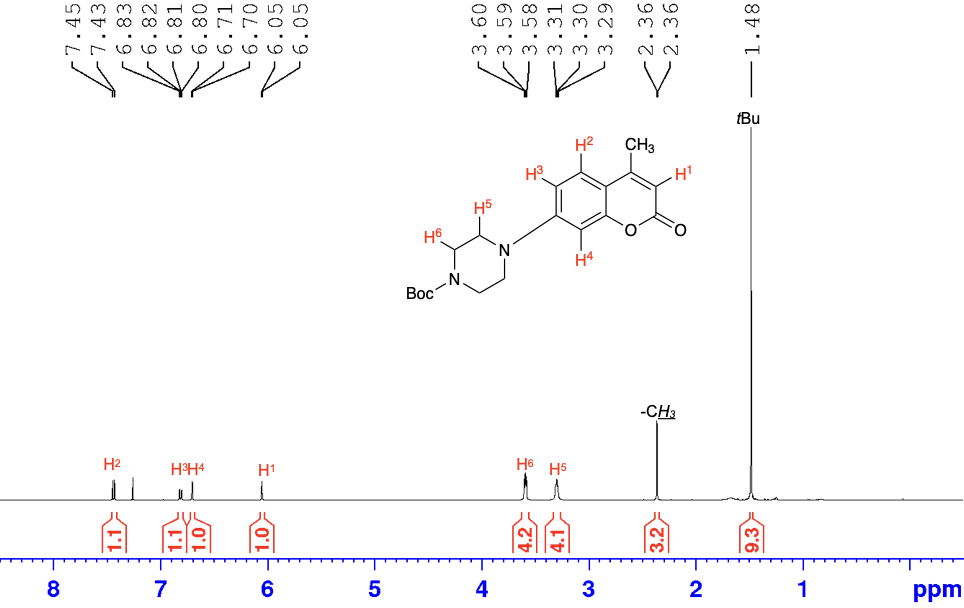
^

Figure S11. ^1^H NMR of compound **16**

**
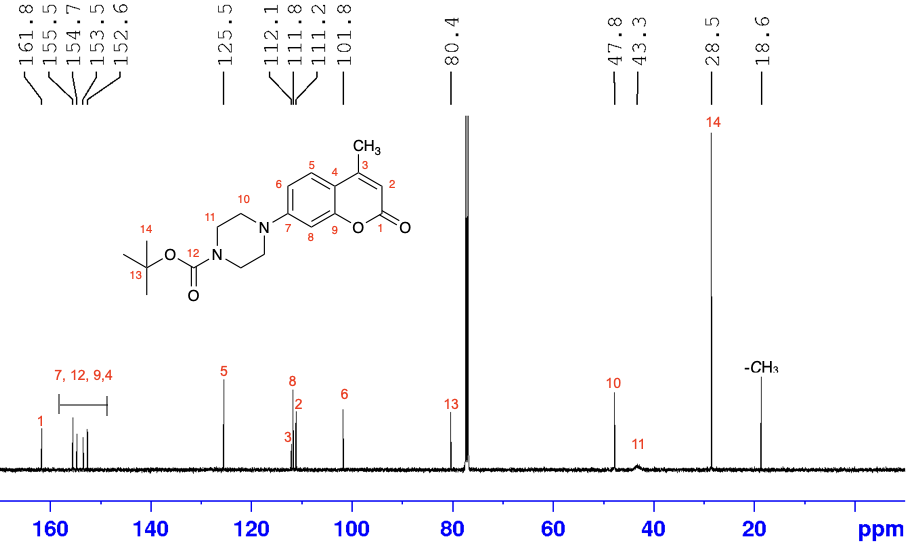
**

Figure S12. ^13^C NMR of compound **16**

^
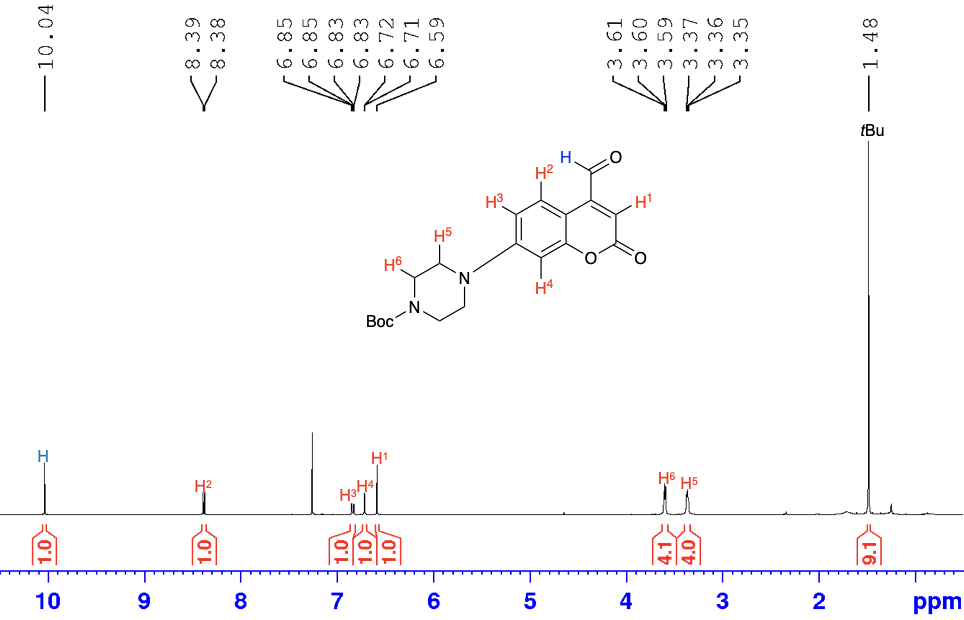
^

Figure S13. ^1^H NMR of compound **17**

**
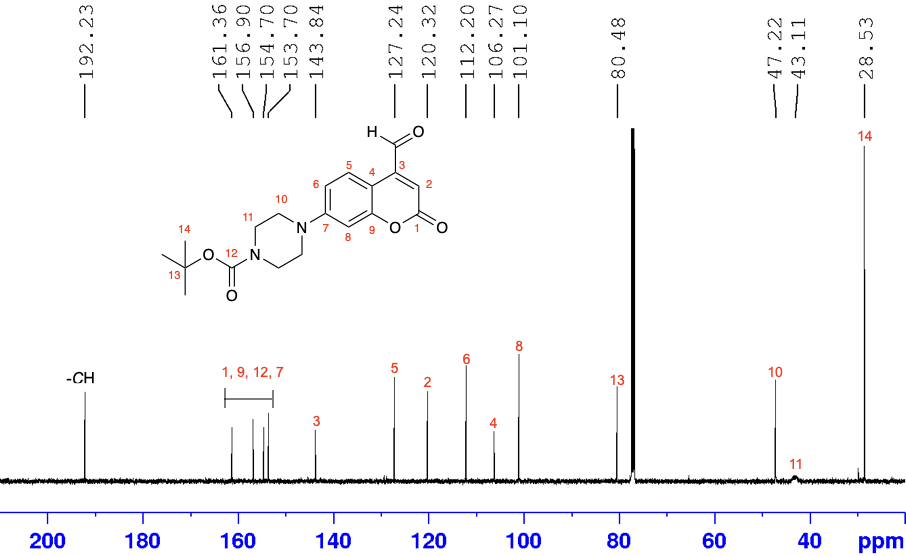
**

Figure S14. ^13^C NMR of compound **17**

^
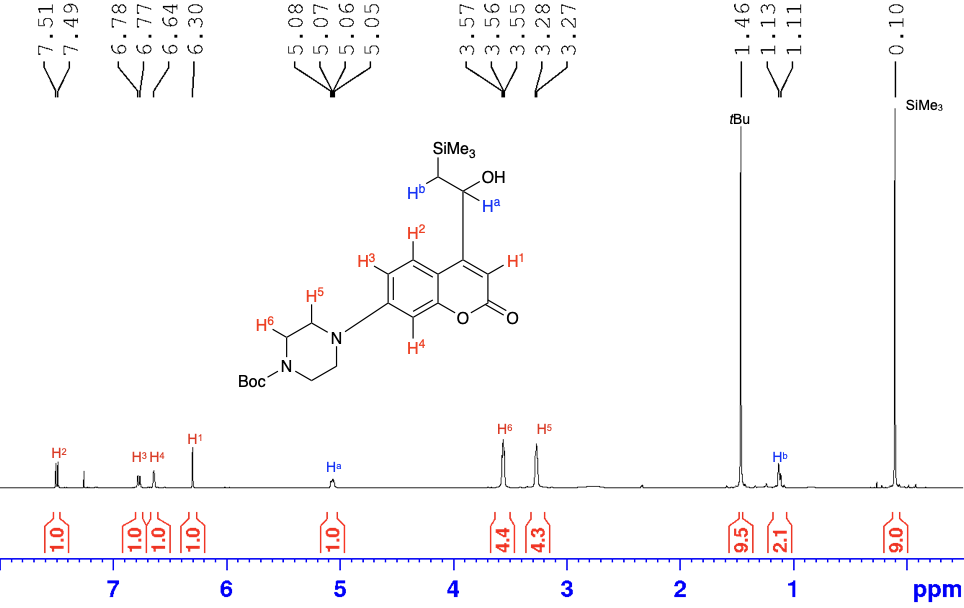
^

Figure S15. ^1^H NMR of compound **18**

^
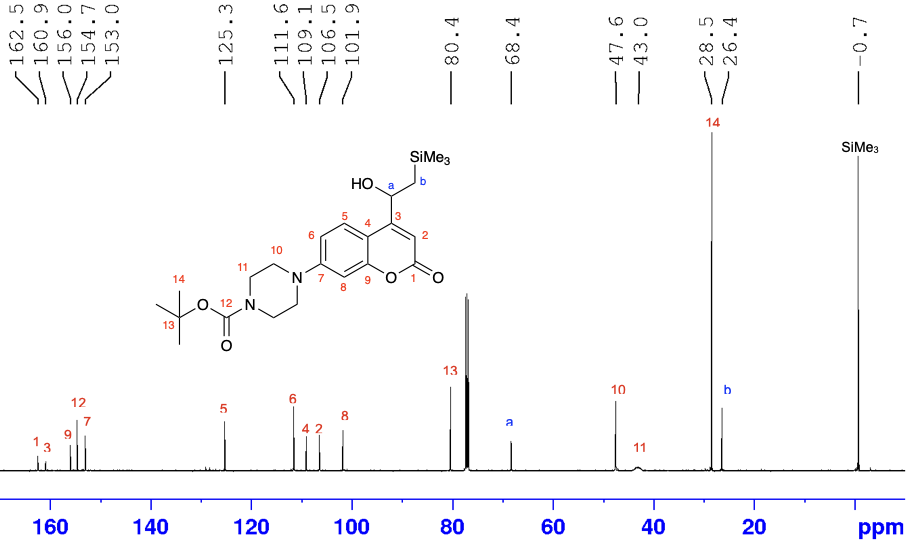
^

Figure S16. ^13^C NMR of compound **18**

^
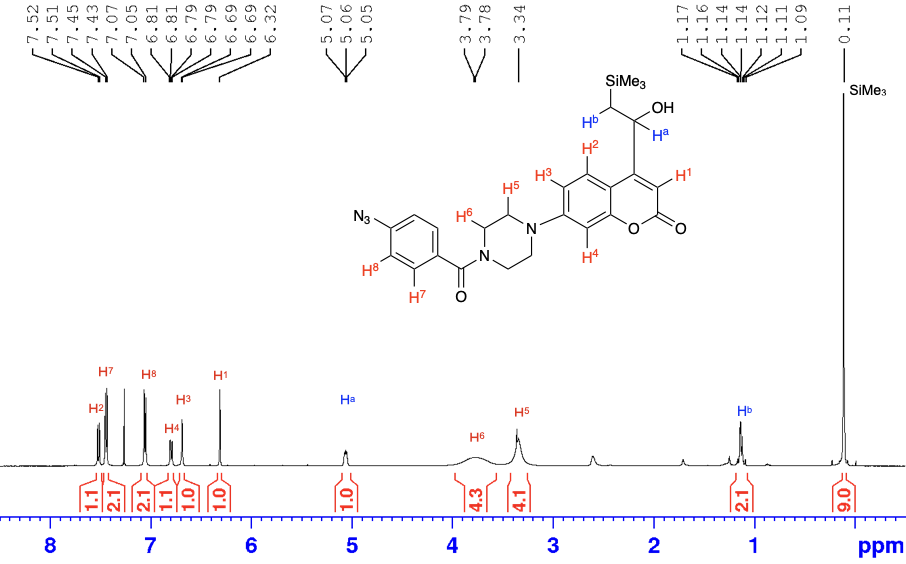
^

Figure S17. ^1^H NMR of compound **19**

^
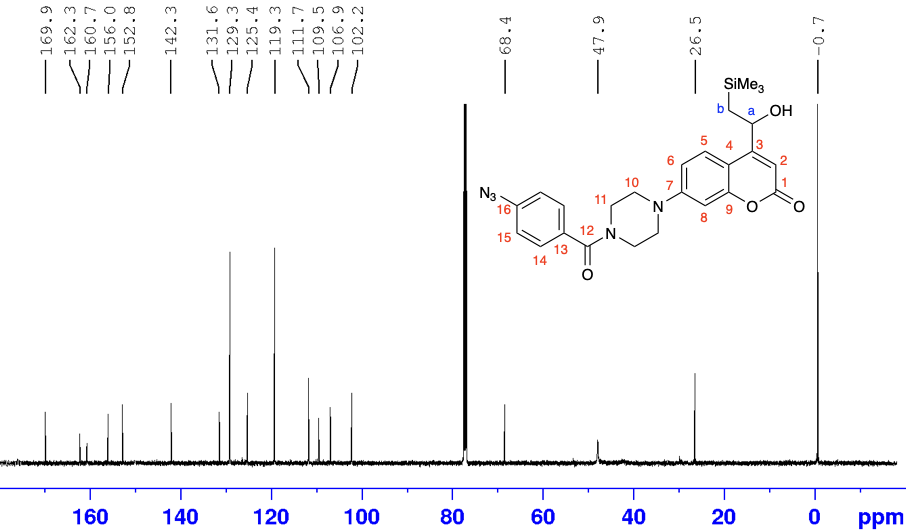
^

Figure S18. ^13^C NMR of compound **19**


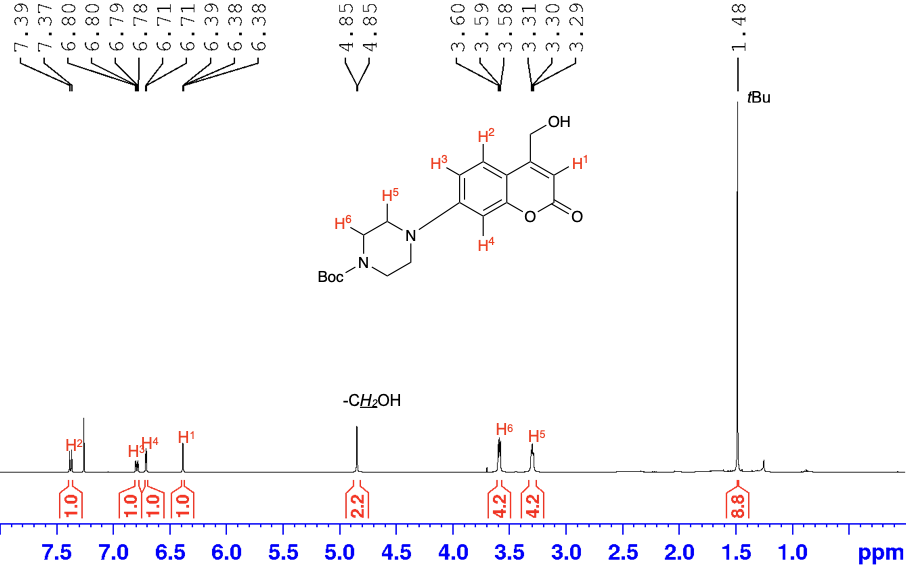


Figure S19. ^1^H NMR of compound **20**

**
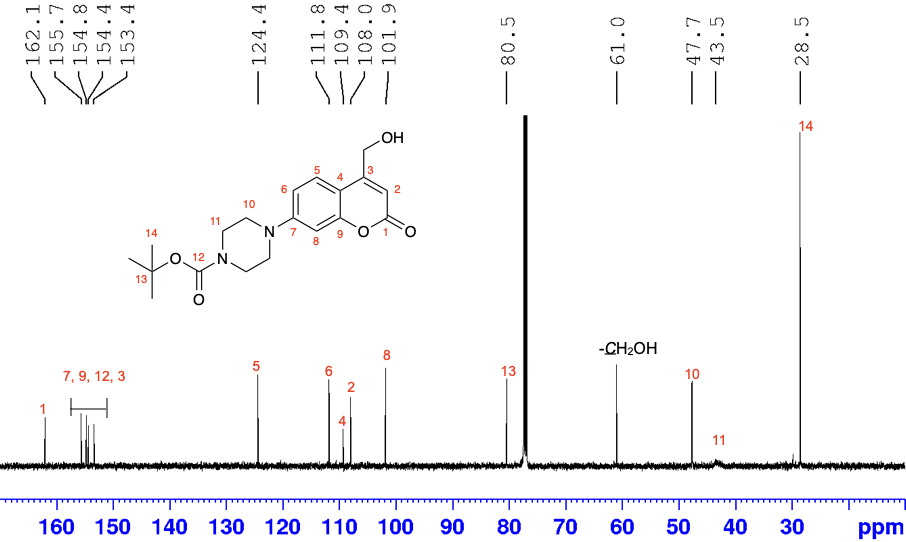
**

Figure S20. ^13^C NMR of compound **20**

^
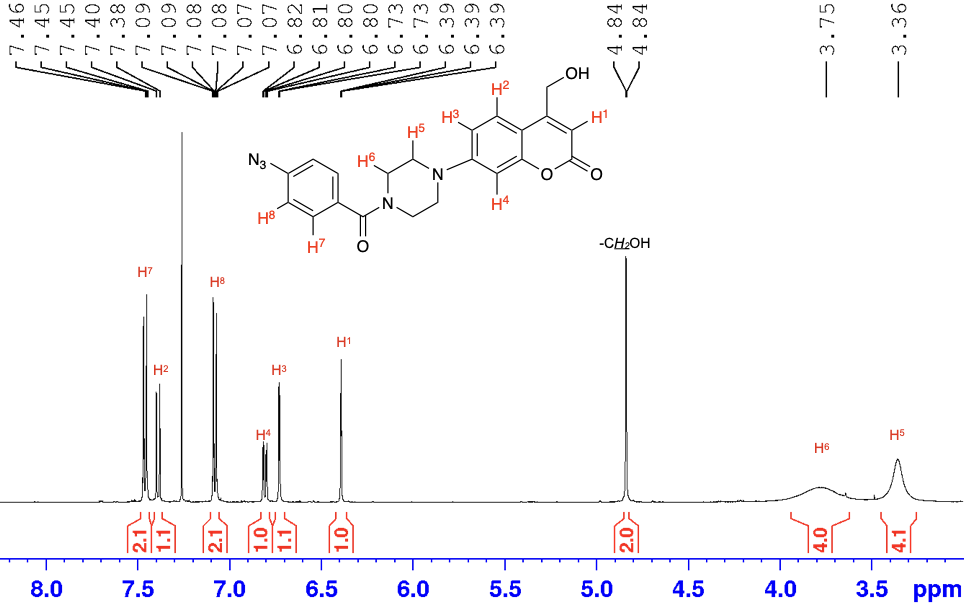
^

Figure S21. ^1^H NMR of compound **21**

**
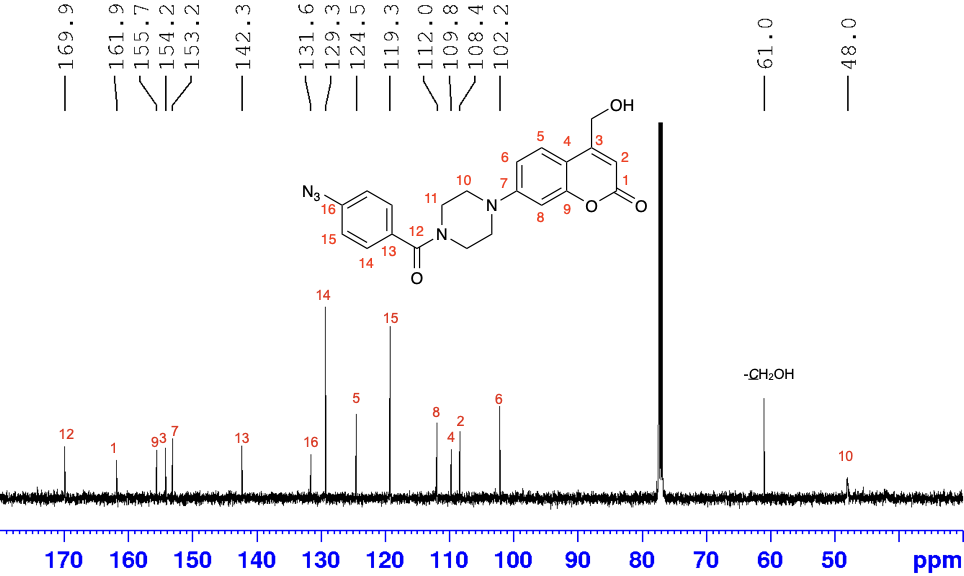
**

Figure S22. ^13^C NMR of compound **21**

^
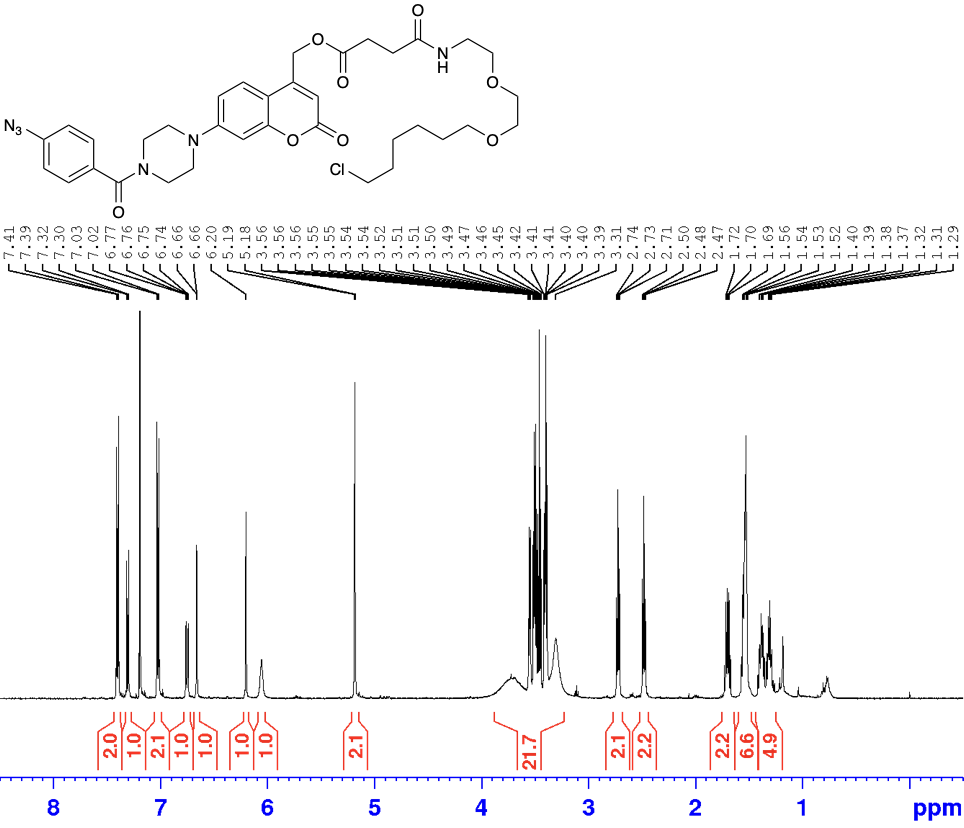
^

Figure S23. ^1^H NMR of compound **7**

**
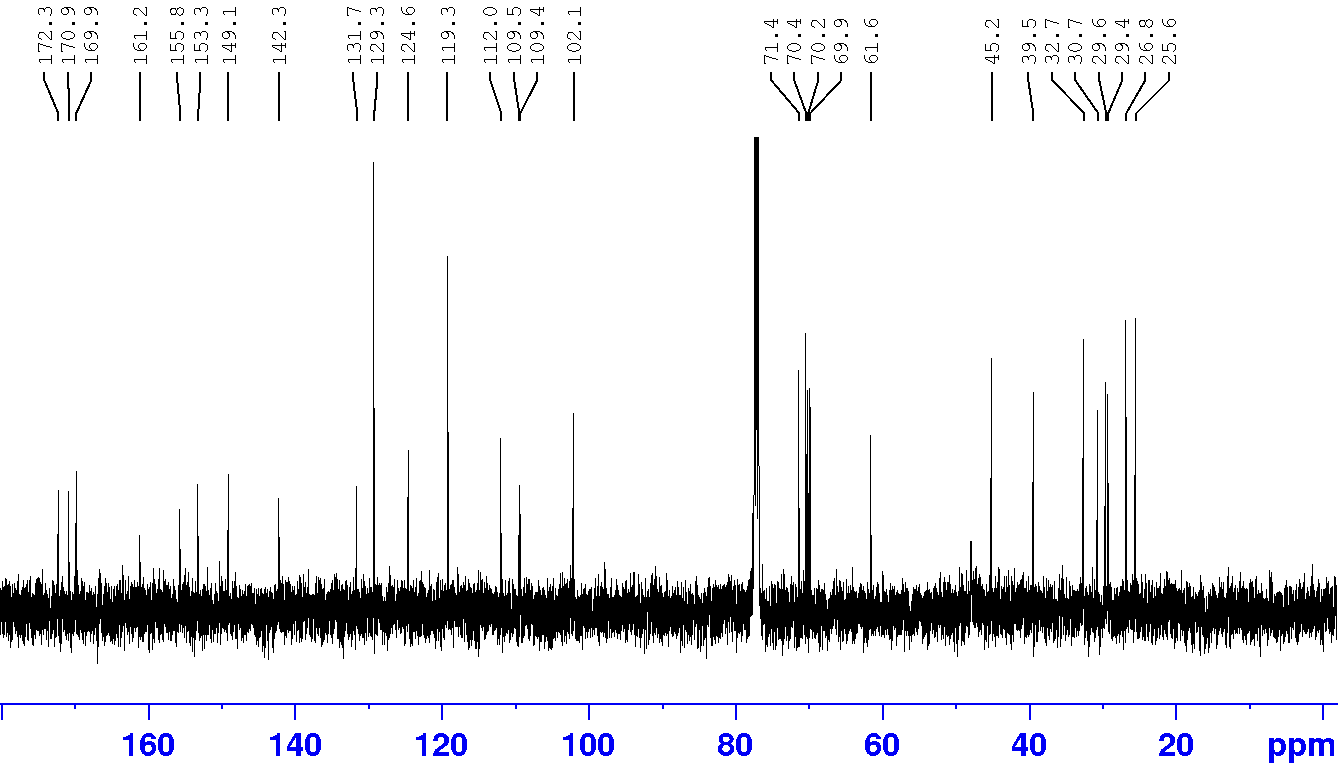
**

Figure S24. ^13^C NMR of compound **7**

^
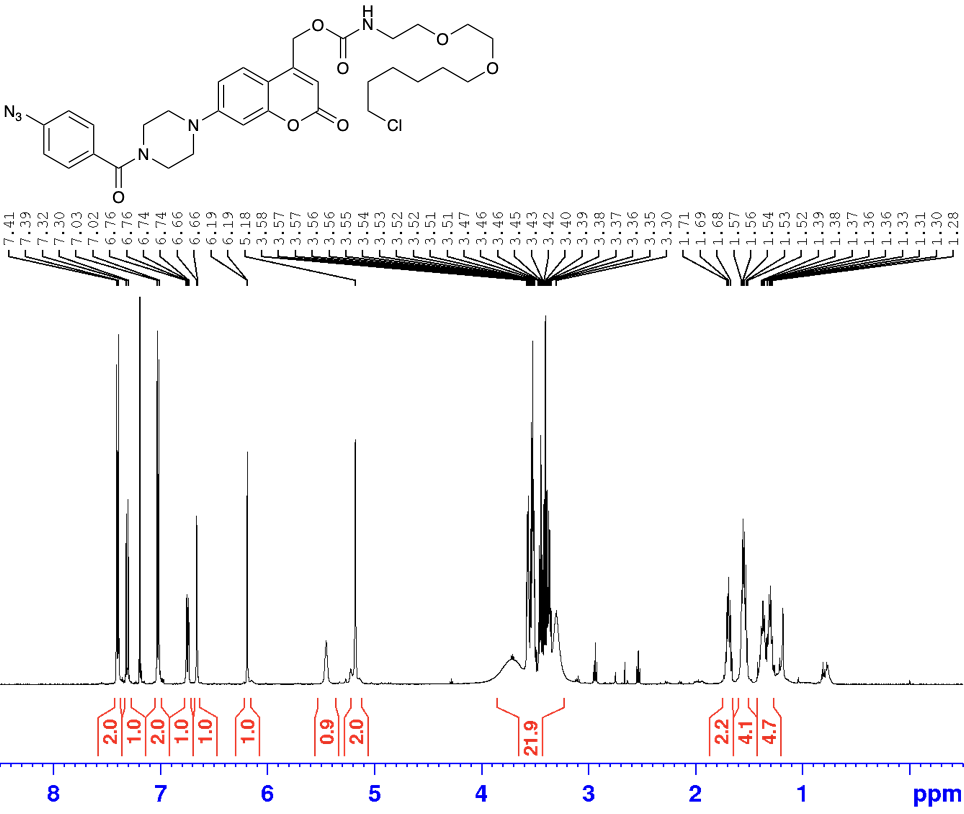
^

Figure S25. ^1^H NMR of compound **8**

**
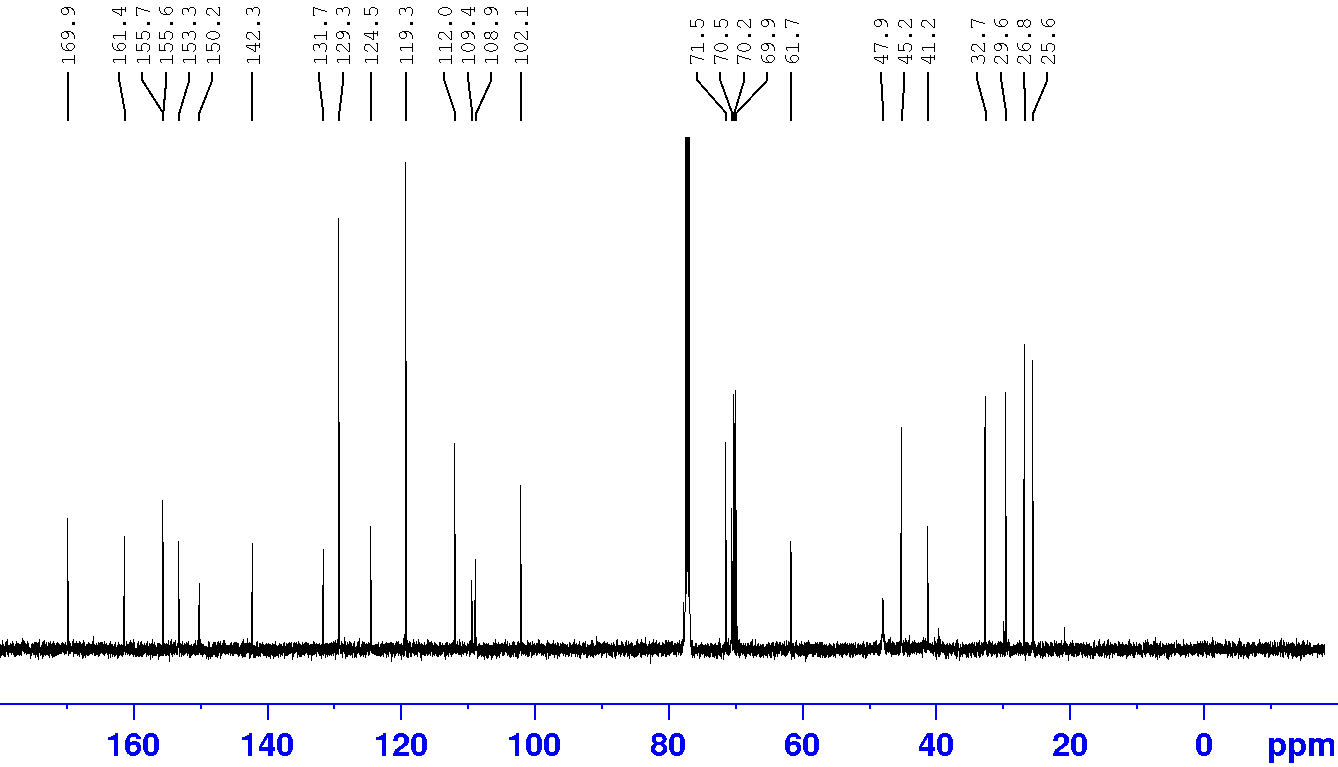
**

Figure S26. ^13^C NMR of compound **8**


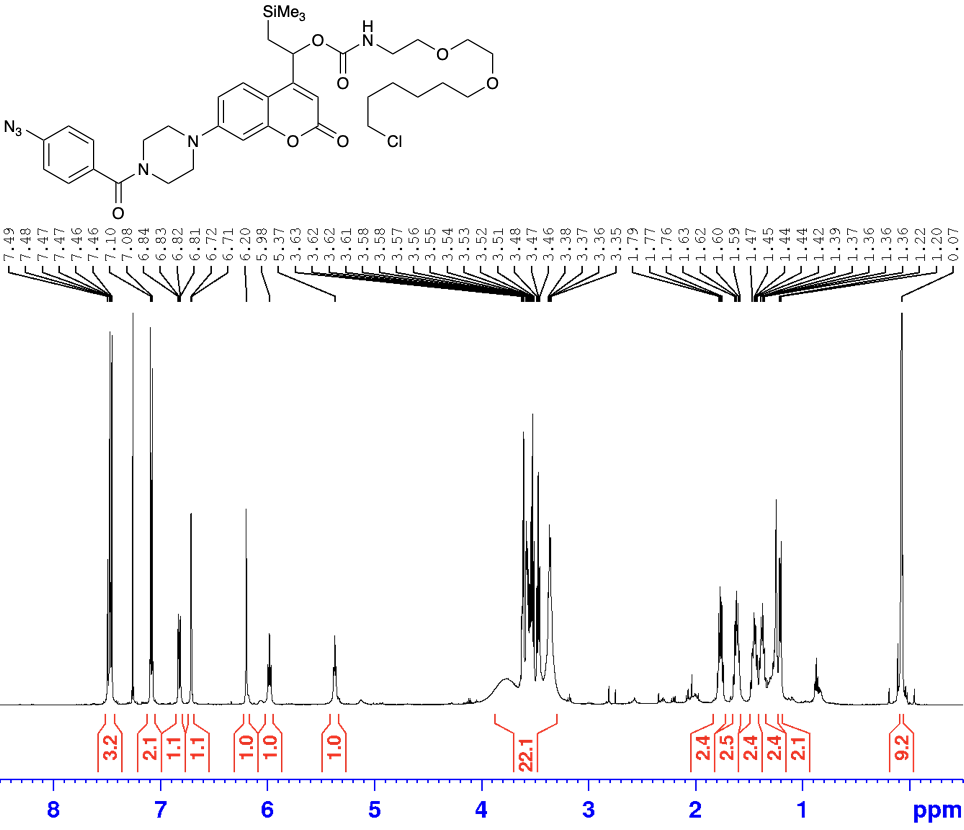


Figure S27. ^1^H NMR of compound **9**

**
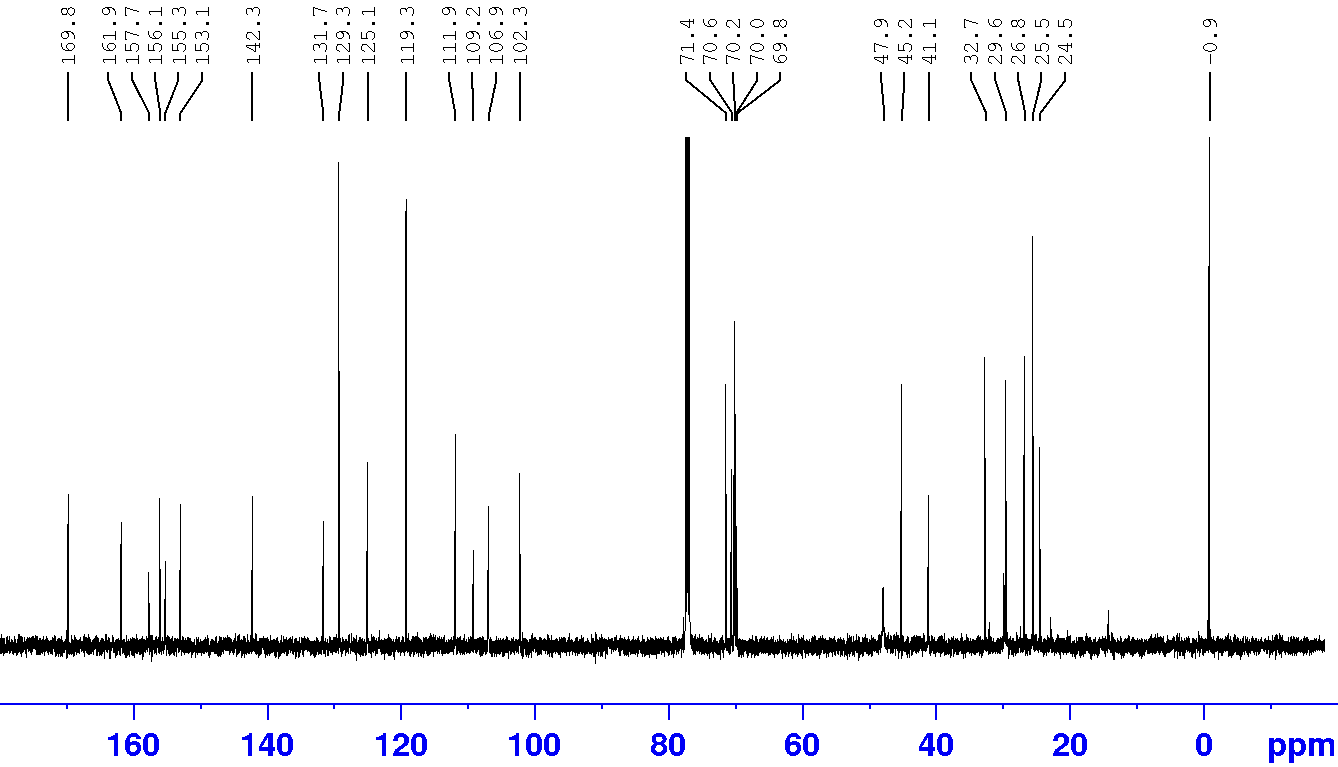
**

Figure S28. ^13^C NMR of compound **9**

**2-2. UV-vis and fluorescence spectra of coumarin derivatives (2, 3, 4, 5)**


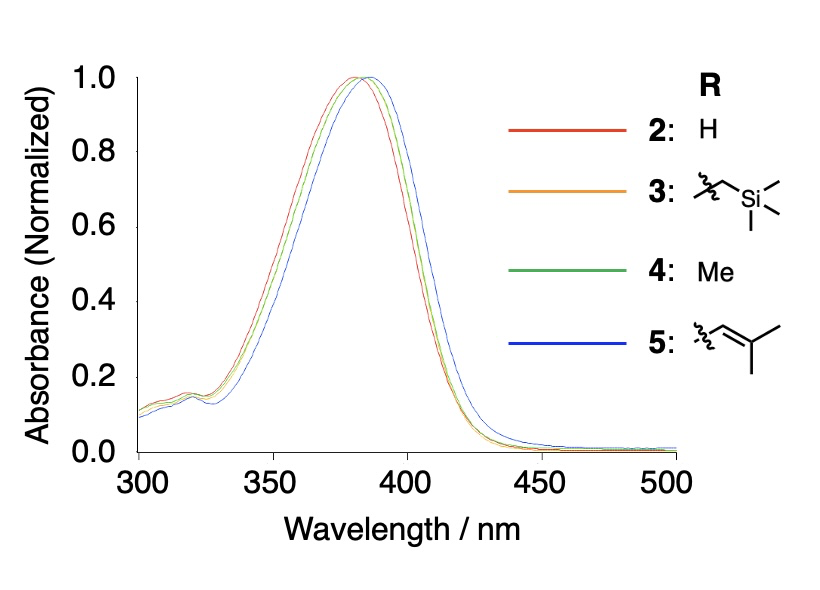


Fig. S29. UV-vis absorption spectra of coumarin derivatives (**2, 3, 4, 5**).


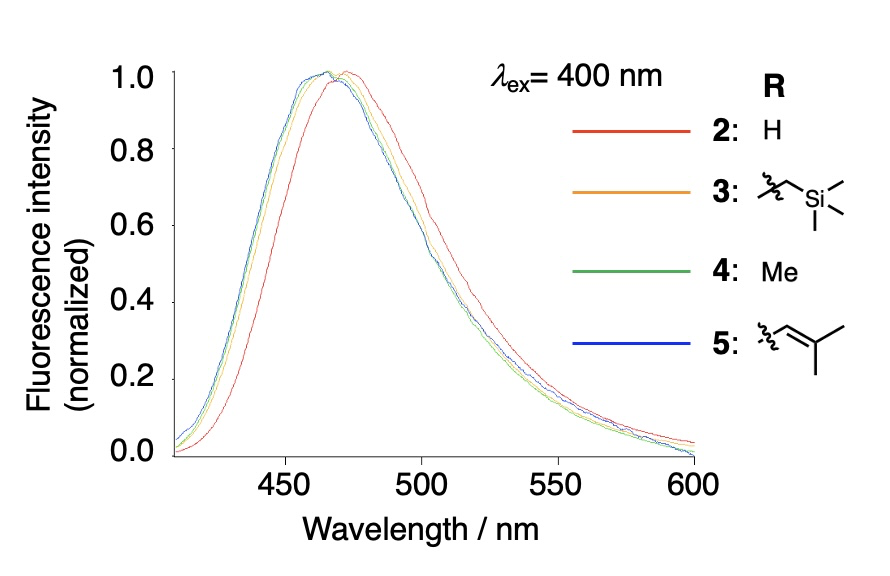


Fig. S30. Fluorescence spectra of coumarin derivatives (**2, 3, 4, 5**).

**2-3. TLC analysis of hydrolysis of coumarins**

**
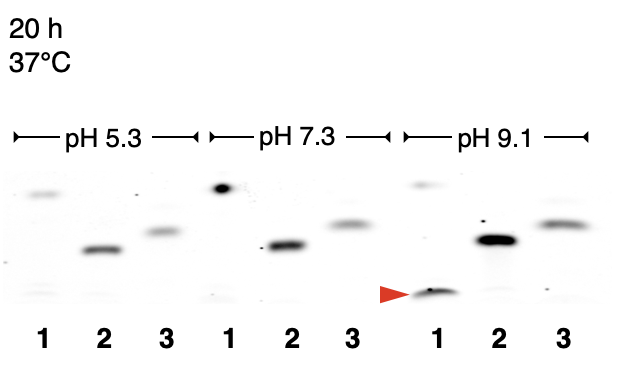
**

Fig. S31. Hydrolytic conversion of coumarin analogs monitored by TLC. Red arrow indicates the hydrolytic product of coumarins.

**2-4. Enzymatic hydrolysis resistance of compound 3 and 5**

**
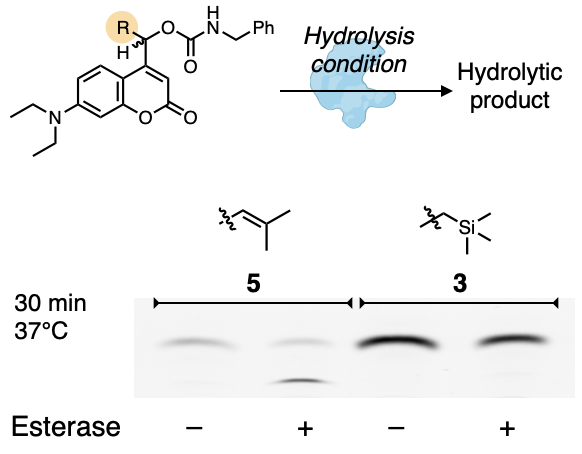
**

Fig. S32. Hydrolytic comversion of compound **3** and **5** in the presence of esterase. Decrease of compound **3** and **5** and generation of the corresponding hydrolytic products were monitored by TLC.

**2-5. HPLC analysis of photolysis of coumarins**

^
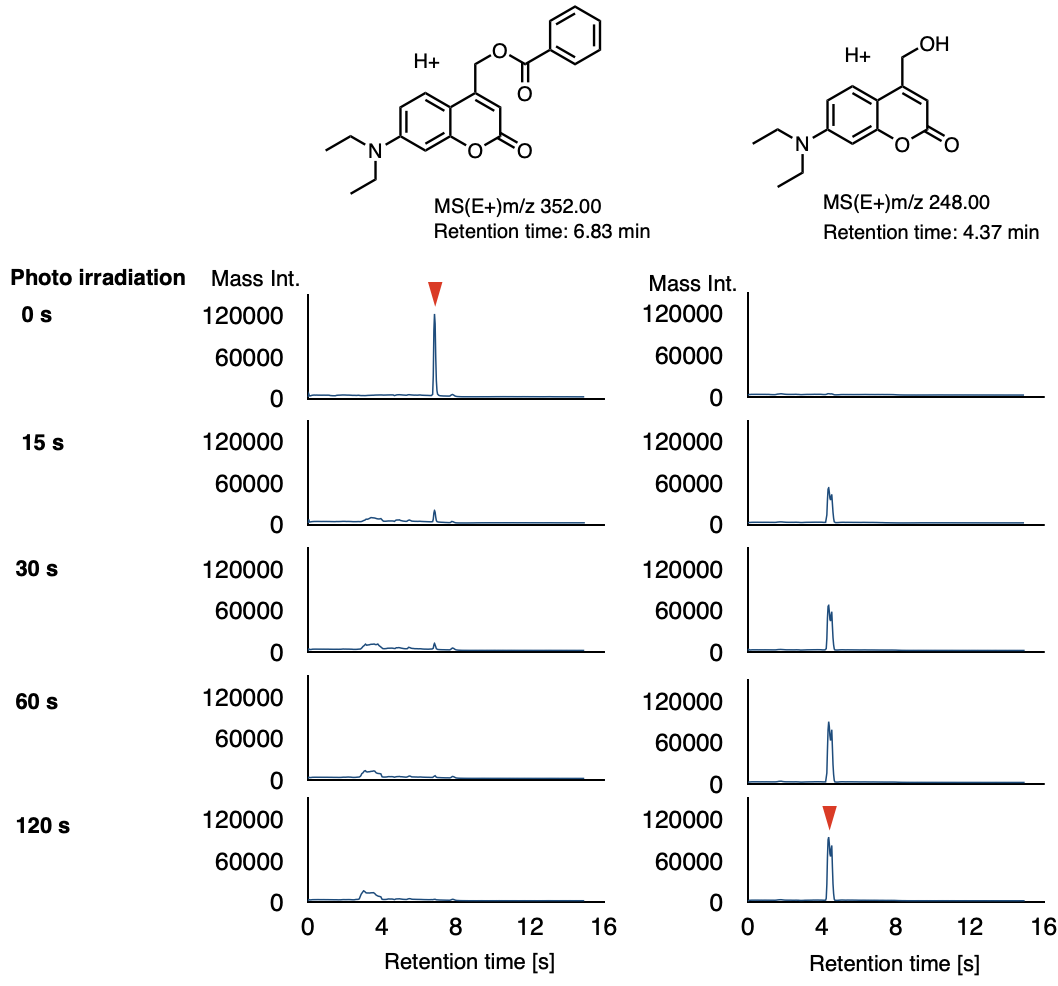
^

Fig. S33. HPLC analysis of photolysis of compound **1**.


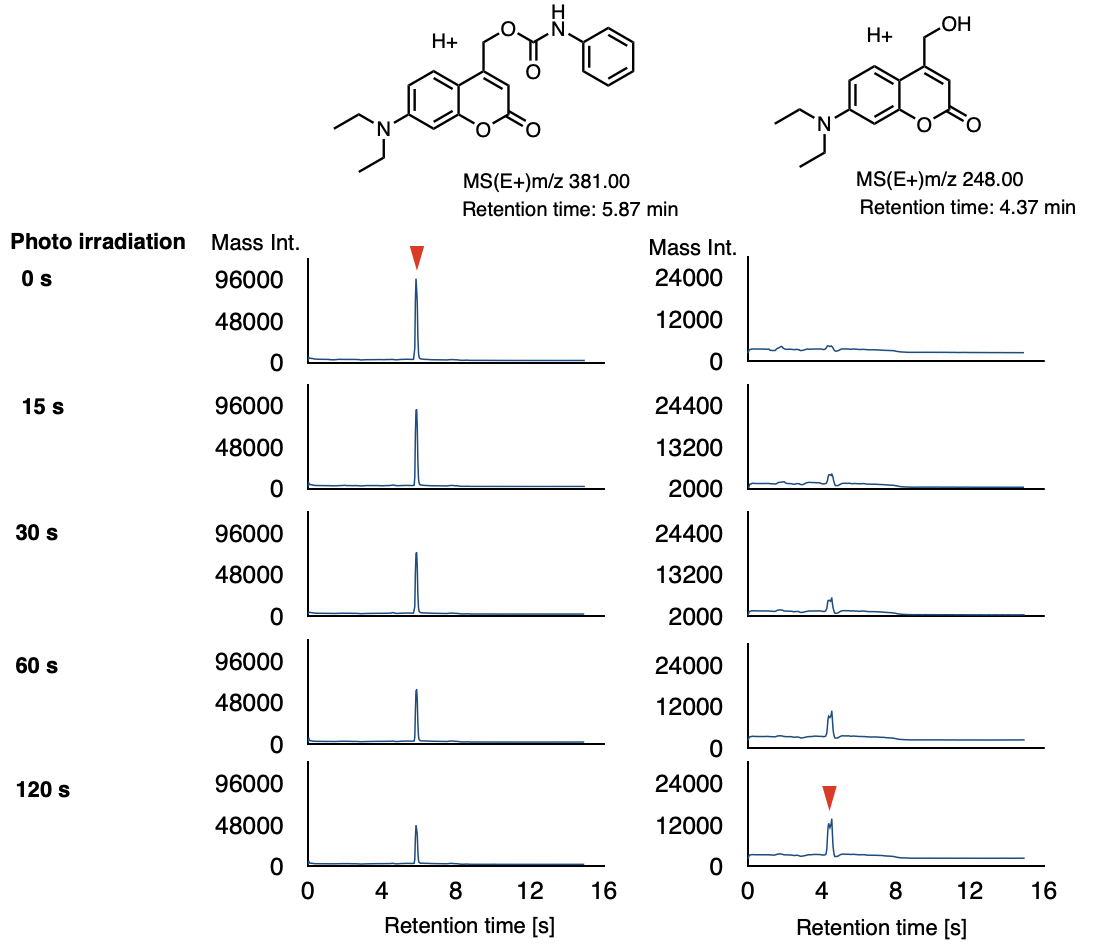


Fig. S34. HPLC analysis of photolysis of compound **2**.


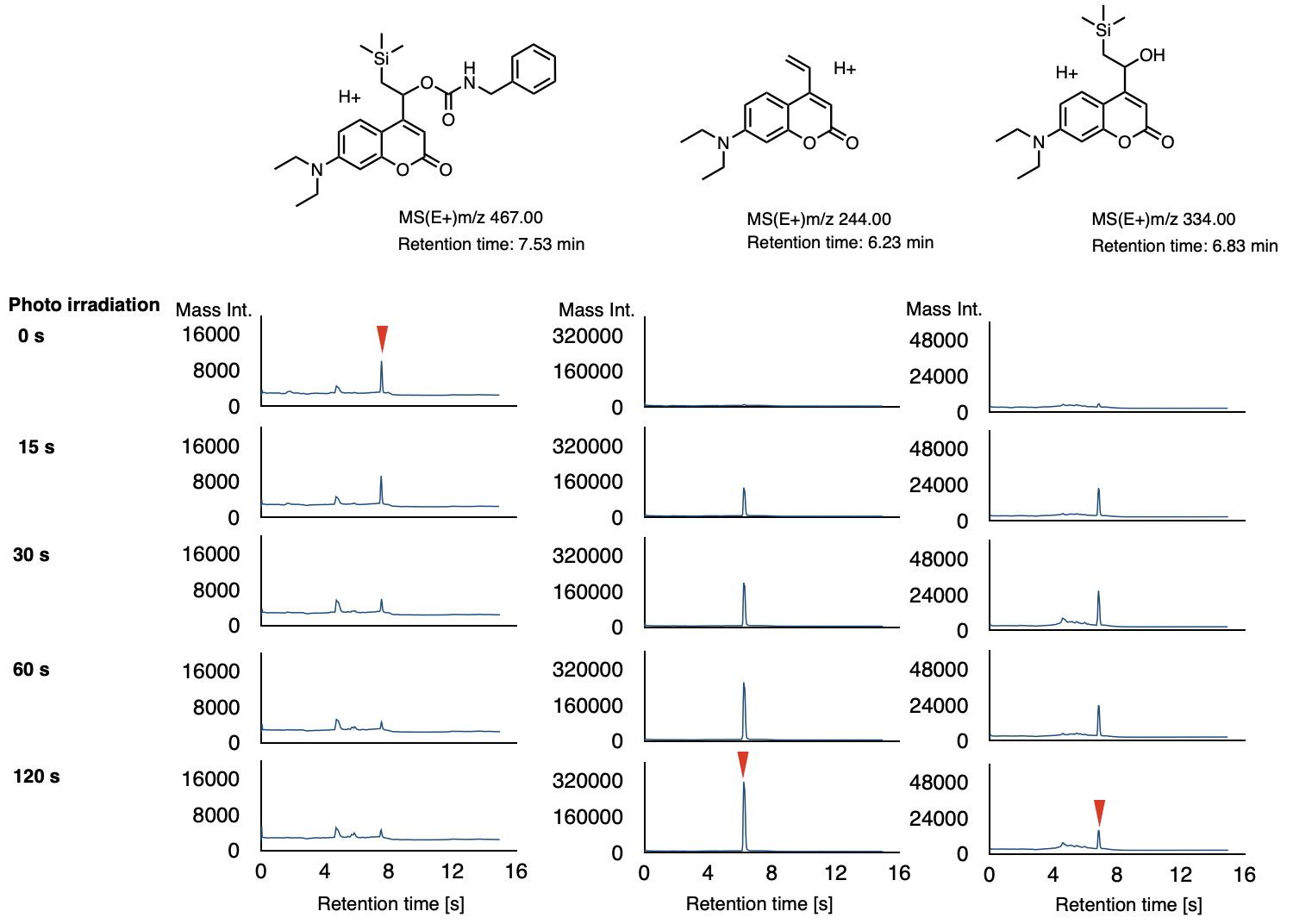


Fig. S35. HPLC analysis of photolysis of compound **3**.

**2-6. Irradiation setup for the photocleavage of coumarin derivatives for ^1^H NMR measurements**

**
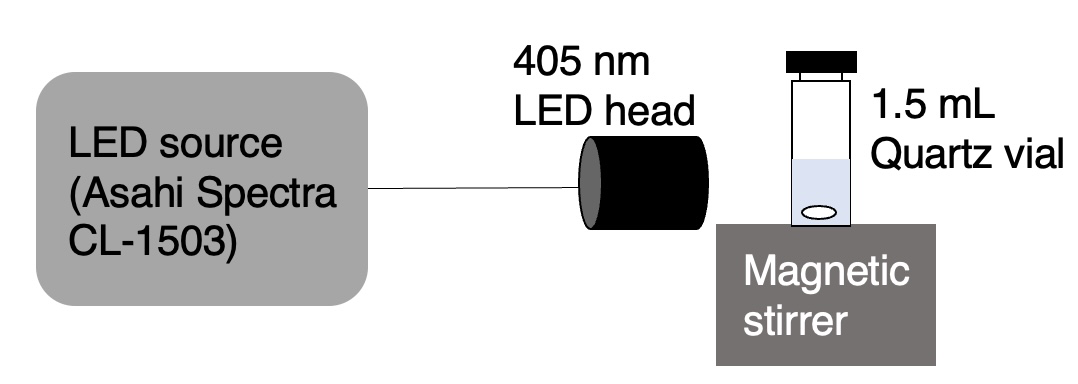
**

Fig. S36. Irradiation setup for photocleavage experiment. The solution for photocleavage was placed in a 1.5 mL quartz vial, ensuring the solution volume was within the light irradiation area, and the reaction was carried out under continuous stirring using a magnetic stirrer during light irradiation. The position of the quartz vial on the magnetic stirrer was fixed throughout the experiment. The light intensity was measured at the stirring position of the coumarin solution using a power meter (Thorlabs PM16-130).

**2-7. Sructural analysis of photolytic product 6**

Mass information of photolytic product **6**

[ C_15_H_17_NO_2_+H]^+^

Measured; m/z 244.1331

Calculated; m/z 244.1332

NMR information of photolytic product **6**

**
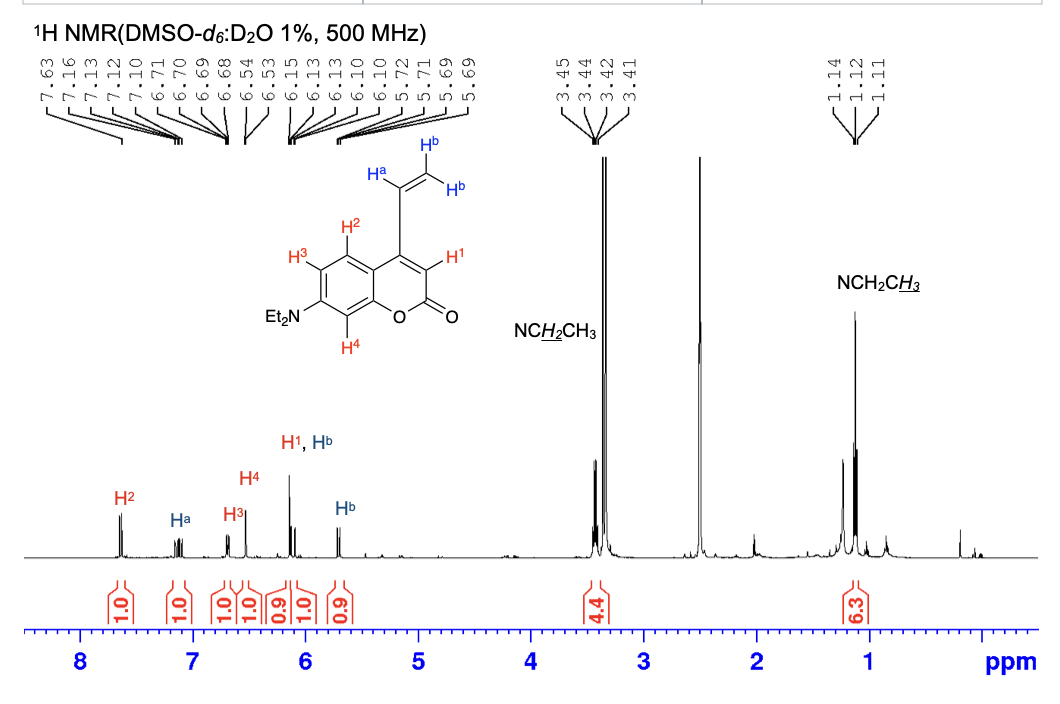
**

Figure S37. ^1^H NMR of compound **6**

**
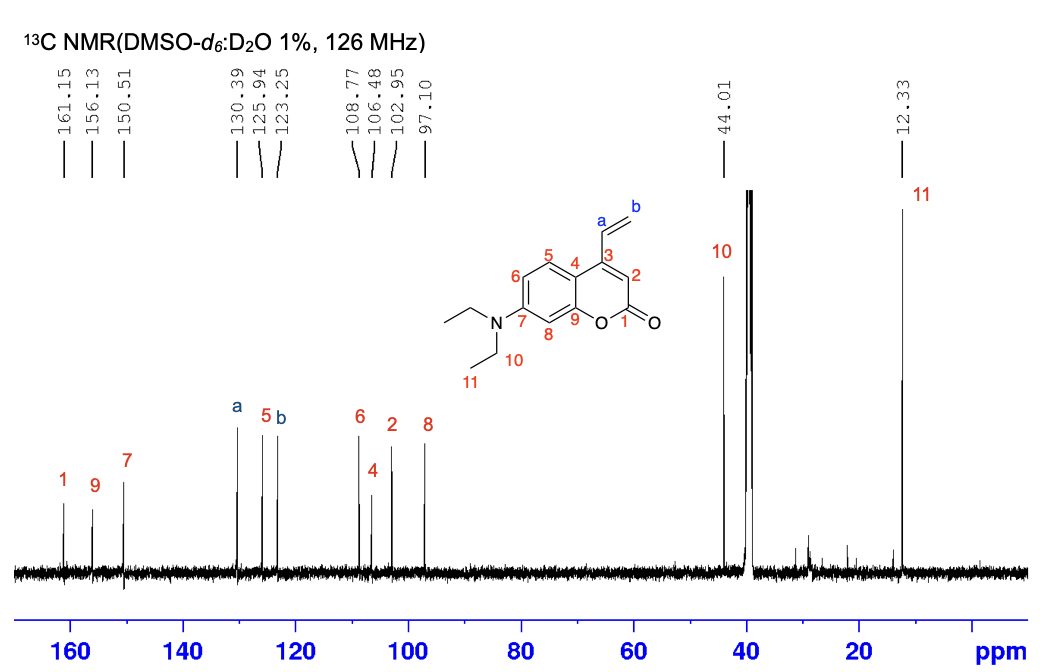
**

Figure S38. ^13^C NMR of compound **6**

**2-8. Plausible mechanism of desilylation upon photocleavage**

^
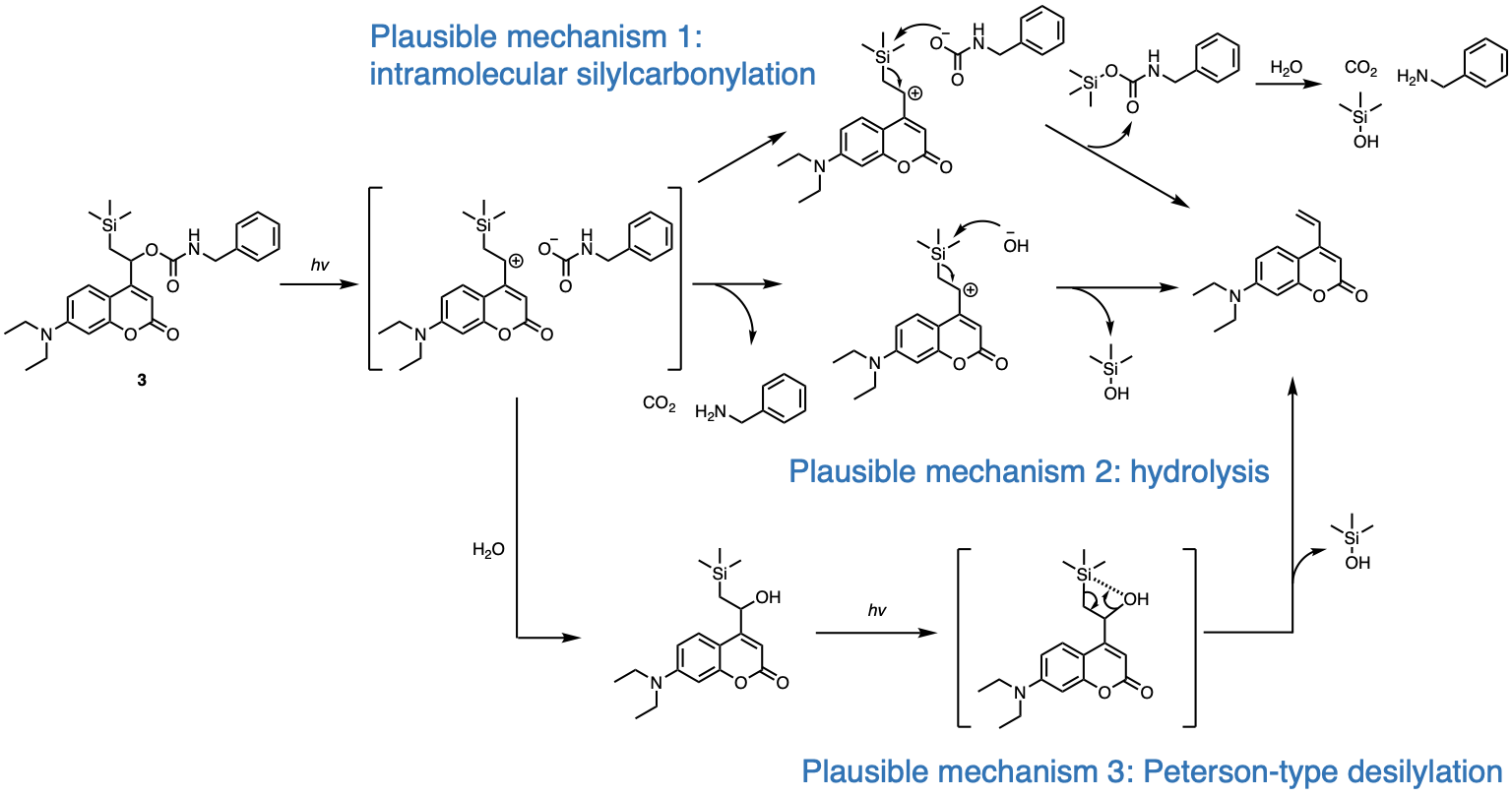
^

Fig. S39. Plausible mechanism of photocleavage of compound **3**.

**2-9. Gel electrophoresis of Halotag conjugated coumarin**

**
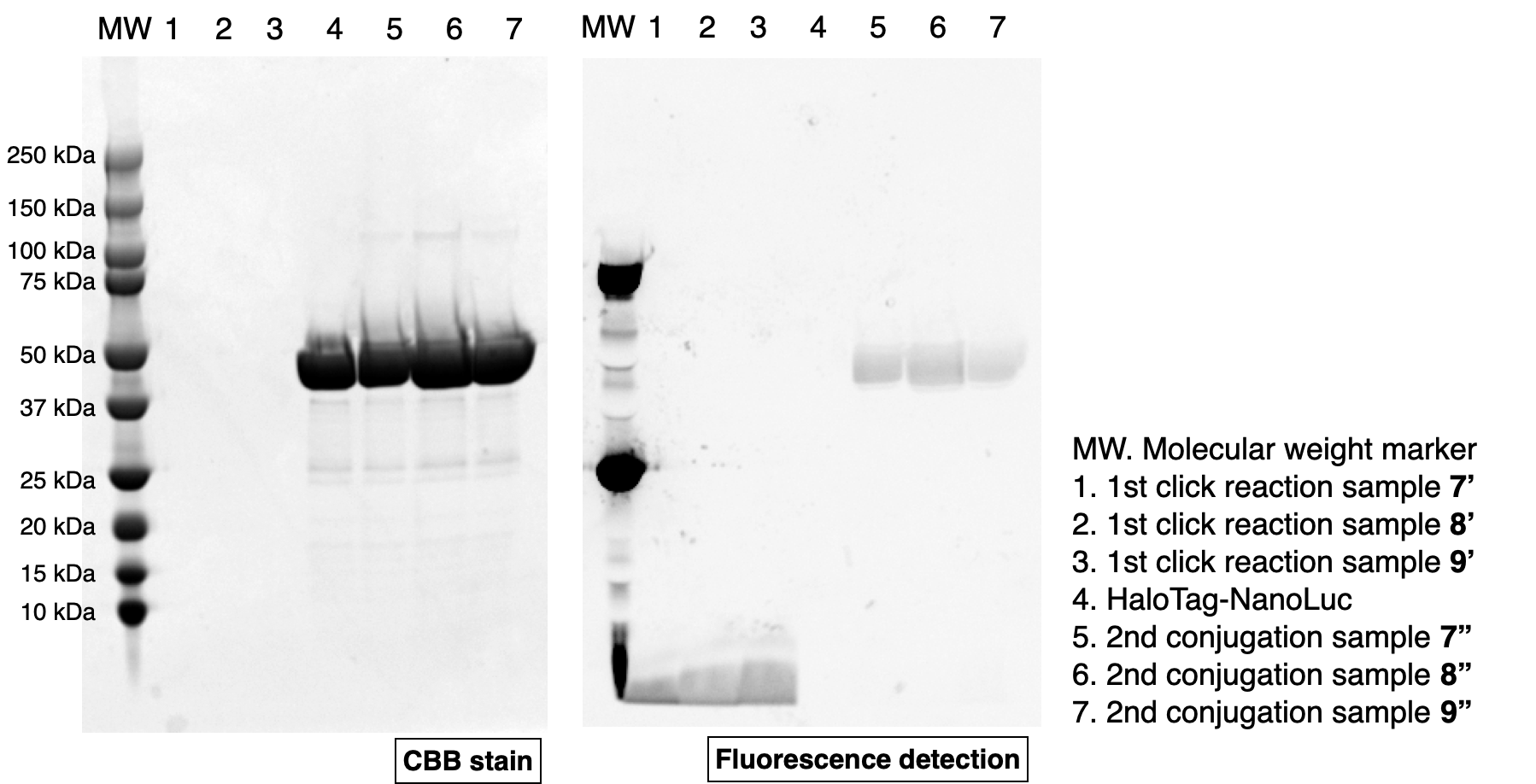
**

Fig. S40. SDS-PAGE analysis of the HaloTag conjugation reaction of biotinylated photolinkers **7’**-**9’**.

1. **Supplementary table**

**Supplementary Table 1**. DNA and protein information of HaloTag-NanoLuc. The affinity purified tag His-Tag is shown in cyan, HaloTag in orange, and NanoLuc in blue. Using vector is pH6HTN.

| **DNA sequence** | **Amino acid sequence** |
| --- | --- |
| ATGAAACATCATCACCATCACCACGCAGAAATCGGTACTGGCTTTCCATTCGACCCCCATTATGTGGAAGTCCTGGGCGAGCGCATGCACTACGTCGATGTTGGTCCGCGCGATGGCACCCCTGTGCTGTTCCTGCACGGTAACCCGACCTCCTCCTACGTGTGGCGCAACATCATCCCGCATGTTGCACCGACCCATCGCTGCATTGCTCCAGACCTGATCGGTATGGGCAAATCCGACAAACCAGACCTGGGTTATTTCTTCGACGACCACGTCCGCTTCATGGATGCCTTCATCGAAGCCCTGGGTCTGGAAGAGGTCGTCCTGGTCATTCACGACTGGGGCTCCGCTCTGGGTTTCCACTGGGCCAAGCGCAATCCAGAGCGCGTCAAAGGTATTGCATTTATGGAGTTCATCCGCCCTATCCCGACCTGGGACGAATGGCCAGAATTTGCCCGCGAGACCTTCCAGGCCTTCCGCACCACCGACGTCGGCCGCAAGCTGATCATCGATCAGAACGTTTTTATCGAGGGTACGCTGCCGATGGGTGTCGTCCGCCCGCTGACTGAAGTCGAGATGGACCATTACCGCGAGCCGTTCCTGAATCCTGTTGACCGCGAGCCACTGTGGCGCTTCCCAAACGAGCTGCCAATCGCCGGTGAGCCAGCGAACATCGTCGCGCTGGTCGAAGAATACATGGACTGGCTGCACCAGTCCCCTGTCCCGAAGCTGCTGTTCTGGGGCACCCCAGGCGTTCTGATCCCACCGGCCGAAGCCGCTCGCCTGGCCAAAAGCCTGCCTAACTGCAAGGCTGTGGACATCGGCCCGGGTCTGAATCTGCTGCAAGAAGACAACCCGGACCTGATCGGCAGCGAGATCGCGCGCTGGCTGTCGACGCTCGAGATTTCCGGCAGCGATAACATGGTCTTTACACTGGAGGATTTCGTCGGGGACTGGGAGCAAACGGCGGCCTACAACTTAGATCAAGTCTTAGAACAAGGCGGTGTCAGCAGCTTATTACAAAACCTTGCGGTTTCCGTAACCCCAATCCAACGCATTGTCCGCTCGGGGGAGAATGCCTTAAAGATTGACATCCACGTCATTATCCCCTATGAGGGACTGTCCGCGGATCAAATGGCACAAATTGAGGAGGTGTTCAAAGTAGTCTATCCCGTGGATGACCACCACTTTAAGGTTATCTTACCATATGGAACGCTTGTAATTGACGGTGTAACCCCCAATATGCTGAACTACTTCGGGCGCCCATACGAGGGCATTGCCGTCTTTGATGGGAAGAAAATTACGGTGACCGGGACCCTGTGGAACGGCAACAAAATCATTGACGAACGTTTAATCACCCCAGATGGATCCATGTTGTTTCGCGTTACAATCAATTCGGGTGTGACTGGTTGGAGACTTTGCGAGAGAATCCTTGCTTAA | MKHHHHHHAEIGTGFPFDPHYVEVLGERMHYVDVGPRDGTPVLFLHGNPTSSYVWRNIIPHVAPTHRCIAPDLIGMGKSDKPDLGYFFDDHVRFMDAFIEALGLEEVVLVIHDWGSALGFHWAKRNPERVKGIAFMEFIRPIPTWDEWPEFARETFQAFRTTDVGRKLIIDQNVFIEGTLPMGVVRPLTEVEMDHYREPFLNPVDREPLWRFPNELPIAGEPANIVALVEEYMDWLHQSPVPKLLFWGTPGVLIPPAEAARLAKSLPNCKAVDIGPGLNLLQEDNPDLIGSEIARWLSTLEISGSDNMVFTLEDFVGDWEQTAAYNLDQVLEQGGVSSLLQNLAVSVTPIQRIVRSGENALKIDIHVIIPYEGLSADQMAQIEEVFKVVYPVDDHHFKVILPYGTLVIDGVTPNMLNYFGRPYEGIAVFDGKKITVTGTLWNGNKIIDERLITPDGSMLFRVTINSGVTGWRLCERILA* |

1. **Abbreviations**

TMS, Trimethylsilyl

DCM, Dichloromethane

NMM, *N*-Methylmorpholine

DMAP, Dimethylaminopyridine

DMF, *N*, *N*-DimethylformamideRT, room temperature

DMSO, Dimethyl sulfoxide

BCN, Bicyclo[6.1.0]non-4-yne

NMR, Nuclear Magnetic Resonance

SDS PAGE, Sodium Dodecyl Sulfate–Polyacrylamide Gel Electrophoresis

TLC, thin layer chromatography

SD, Standard Deviation.

1. **Supplementary references**
2. A. M. Schulte, G. Alachouzos, W. Szymanski, B. L. Feringa, *J. Am. Chem. Soc.* **2022**, *144*, 12421.
3. A. Hofer, G. S. Cremosnik, A. C. Müller, R. Giambruno, G. Trefzer, G. Superti-Furga, K. L. Bennett, H. J. Jessen, *Chem. Eur. J.* **2015**, *21*, 10116.
4. J. I. Müller, K. Kusserow, G. Hertrampf, A. Pavic, J. Nikodinovic-Runic, T. A. M. Gulder, *Org. Biomol. Chem.* **2019**, *17*, 1966.
